# Progress in research on RAAS inhibitors regulating inflammatory responses and oxidative stress: from molecular mechanisms to clinical evidence

**DOI:** 10.3389/fphar.2026.1925933

**Published:** 2026-10-15

**Authors:** Hongtao Yin, Mengdi Yang, Shiming Zhang, Qiong Li, Donghu Zhen, Xulei Tang

**Affiliations:** 1 Department of Endocrinology, The First Hospital of Lanzhou University, Lanzhou, China; 2 The First Clinical Medical College, Lanzhou University, Lanzhou, China; 3 Department of Cardiac Surgery, The First Hospital of Lanzhou University, Lanzhou, China

**Keywords:** ACE2-Ang-(1–7)-mas axis, inflammatory response, oxidative stress, RAAS inhibitors, target organ protection

## Abstract

The renin-angiotensin-aldosterone system (RAAS) inhibitors serve as cornerstone pharmacotherapies for chronic conditions including hypertension, heart failure, and chronic kidney disease. Accumulating evidence over recent years has demonstrated that their target organ-protective effects cannot be fully attributed to blood pressure-lowering actions alone; instead, the modulation of inflammatory responses and oxidative stress emerges as a core mechanism underpinning their extra-antihypertensive benefits. However, systematic comparisons of such effects across distinct classes of RAAS inhibitors and integration of the latest evidence remain lacking. This review focuses on the molecular regulatory network of the dual RAAS axes, systematically dissecting the specific molecular mechanisms by which the canonical ACE-AngII-AT1R axis mediates tissue injury—via activation of the NLRP3 inflammasome, NADPH oxidase, and NF-κB signaling pathways—while the protective ACE2-Ang(1–7)-MasR axis exerts anti-inflammatory and antioxidant effects. A cross-class comparison is conducted to evaluate the divergent effects of five clinically prevalent RAAS inhibitor classes (ACE inhibitors [ACEI], angiotensin receptor blockers [ARB], direct renin inhibitors [DRI], angiotensin receptor-neprilysin inhibitors [ARNI], and mineralocorticoid receptor antagonists [MRA]) on the regulation of inflammation and oxidative stress. By synthesizing the latest evidence from basic science and clinical studies over the past decade, we summarize the distinct organ-protective profiles of these agents across various target organs affected by cardiovascular disease, chronic kidney disease, pulmonary disorders, neurological conditions, rheumatoid arthritis, and osteoporosis. Finally, addressing the limitations and inconsistencies within existing literature, this article proposes future research directions, strategies for optimizing combination therapies, and refined clinical trial designs to enhance the clinical application of RAAS inhibitors and facilitate the development of novel organ-protection paradigms.

## 1 Introduction

Cardiovascular disease has emerged as one of the leading causes of global mortality. As shared core pathophysiological mechanisms underlying multiple diseases, inflammatory responses and oxidative stress play a pivotal role in the pathogenesis and progression of cardiovascular disorders. While the RAAS was traditionally regarded as primarily responsible for blood pressure regulation and water-electrolyte metabolism, a growing body of evidence has confirmed the existence of a complex reciprocal regulatory relationship between the RAAS and inflammatory responses/oxidative stress (Ekholm and Kahan, 2021). Angiotensin II (Ang II), the central effector molecule of the renin-angiotensin-aldosterone system (RAAS), induces the expression of pro-inflammatory cytokines and the generation of reactive oxygen species (ROS) via multiple signaling pathways, thereby contributing to tissue injury and disease progression (Cantero-Navarro et al., 2021). In the pathological processes of diverse conditions—such as atherosclerosis, hypertension, heart failure, diabetic nephropathy, and arthritis—excessive RAAS activation, elevated inflammatory factor levels, and enhanced oxidative stress are consistently observed (Ekholm and Kahan, 2021; Dwivedi and Sikarwar, 2025; Habib et al., 2025). Grounded in this pathophysiological basis, RAAS inhibitors, including angiotensin-converting enzyme inhibitors (ACEIs), angiotensin II receptor blockers (ARBs), direct renin inhibitors (DRIs), and mineralocorticoid receptor antagonists (MRAs), have been widely employed in the clinical management of cardiovascular and renal diseases. Notably, their cardioprotective effects are robustly supported by a large body of evidence-based medical data (Stolfo and Savarese, 2019; Te et al., 2015).

However, the pleiotropic mechanisms of RAAS inhibitors—particularly the specific molecular pathways underlying their anti-inflammatory and antioxidant effects, as well as their clinical implications—have not yet been fully elucidated. Controversy persists regarding the relative anti-inflammatory and antioxidant efficacy of distinct classes of RAAS inhibitors, and preclinical findings do not always align perfectly with clinical observations. With the continuous advancement of basic research and the accumulation of clinical evidence, the potential applications of RAAS inhibitors beyond cardiovascular and renal diseases are increasingly being uncovered in fields such as metabolic syndrome, pulmonary diseases, neurological disorders, and bone metabolism (Armstrong et al., 2026). Therefore, systematically synthesizing the research progress on the interplay between RAAS inhibitors, inflammatory responses, and oxidative stress not only facilitates a deeper understanding of the mechanisms of action of these agents but also provides a scientific foundation for expanding their clinical utility. This review aims to comprehensively summarize advances in the study of RAAS inhibitors in modulating inflammatory responses and oxidative stress, spanning mechanistic investigations, clinical evidence, and meta-analyses (Figure 1), with the objective of providing a reference for rational clinical medication and an in-depth understanding of drug mechanisms (Table 1).

**FIGURE 1:**
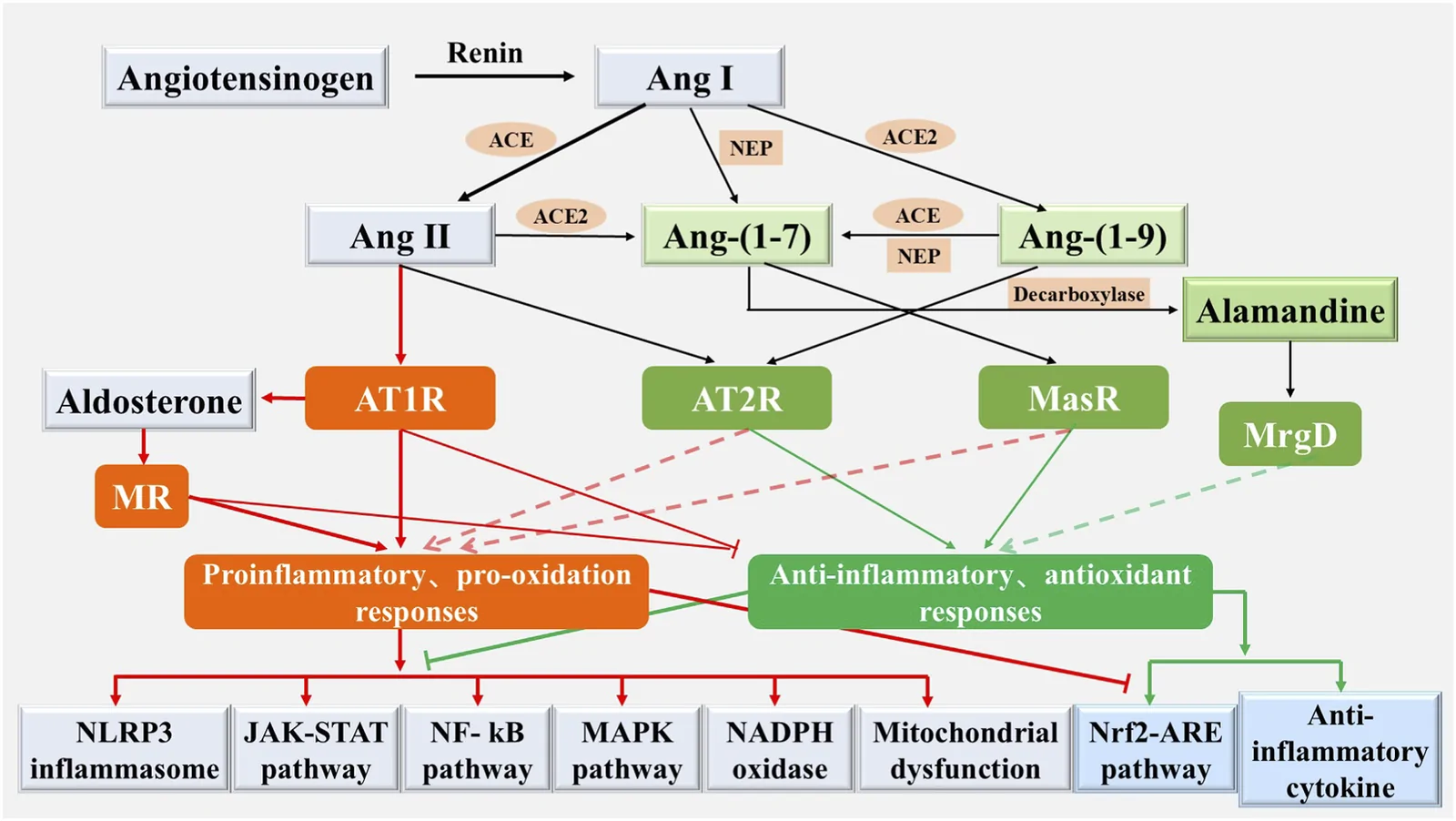
Overview of the Mechanistic Links Between the Renin-Angiotensin System (RAAS) and Inflammation, Oxidative Stress. Key bioactive molecules within the renin-angiotensin system (RAAS) and their major or most widely recognized effects on inflammation and oxidative stress are depicted in this figure. Early research predominantly focused on the pro-inflammatory and pro-oxidative actions of angiotensin II (Ang II) via activation of the AT1 receptor (AT1R) and aldosterone. In recent years, protective RAAS pathways, including the ACE2/Ang 1–7/Mas axis and the ACE2/Ang-(1–9)/AT2R axis, have increasingly attracted attention; preclinical studies have confirmed their anti-inflammatory and antioxidant effects, though the translational relevance of these findings to humans has yet to be evaluated. Most recently, the mechanistic roles of the Mas-related G protein-coupled receptor D (MrgD) and the Ang-(1–7)/alamandine/MrgD axis are also being elucidated. Red lines denote pro-inflammatory and pro-oxidative responses, while green lines indicate anti-inflammatory and antioxidant responses.

**TABLE 1:** Comparative analysis of anti-inflammatory and antioxidant potencies among different classes of RAAS inhibitors.

Classes of Medications	Target Receptor	Primary Molecular Target	Key Downstream Signaling Pathways (Core Anti-inflammatory and Antioxidant Mechanisms)	Anti-inflammatory/Antioxidant Efficacy (Rating)	Major Target Organs	Key Clinical Limitations
ACEI	ACE (a zinc metalloprotease, somatic type with C/N two catalytic domains; non-specific, degrading both bradykinin and substance P simultaneously)	Inhibits angiotensin-converting enzyme (ACE), blocking the conversion of Ang I to Ang II; reduces bradykinin degradation → increases bradykinin/B2 receptor signaling	1. ↓NF-κB (inhibits IκBα phosphorylation/degradation, preventing p65/p50 nuclear translocation) 2. ↓Nox1/Nox2/Nox4 → ROS 3. ↑Nrf2/Keap1 dissociation → ARE → HO-1/NQO1/SOD/GPx 4. ↑Bradykinin → B2R → PLCβ → eNOS → NO → sGC → cGMP → PKG 5. Limited evidence for direct activation of PPARγ	★★★	Cardiovascular, renal, and vascular systems (robust clinical evidence); central nervous system and pulmonary systems (predominantly preclinical data, with limited clinical evidence	Dry cough, angioedema (attributable to bradykinin accumulation); incomplete suppression of Ang II due to chymase-mediated escape; hyperkalemia; teratogenicity and contraindication in
ARB	AT1R (GPCR coupled to Gq/11) AT2R is a counter-regulatory receptor	Selectively blocks the AT_1_ receptor, preventing Ang II from binding to AT1R and inhibiting the Gq/11–PLCβ signaling pathway; relieves the inhibition of AT2R by AT1R	1. ↓NF-κB 2. ↓Nox→ROS 3. ↑Nrf2/ARE 4. Telmisartan exhibits partial PPARγ agonist activity (other ARBs show inconsistent evidence) 5. AT2R activation partially mediates protective effects	★★★	Cardiovascular, renal, and vascular systems (supported by substantial clinical evidence); central nervous system and pulmonary systems (evidence derived primarily from preclinical studies)	Reflex elevation of Ang II; escape from tissue RAAS suppression; hyperkalemia; teratogenicity and contraindication in pregnancy; lower incidence of dry cough and angioedema compared with ACE inhibitors
DRI	Renin (aspartyl protease, substrate is angiotensinogen AGT)	Binds to the renin active site, directly inhibits renin activity, and blocks the cleavage of angiotensinogen into Ang I	1. ↓NF-κB 2. ↓Nox→ROS 3. ↓TGF-β/Smad 4. ↑Nrf2 (evidence relatively limited, primarily preclinical)	★	Renal, cardiac, and vascular systems (with limited evidence for target organ hard endpoints)	Effective in blood pressure reduction, but evidence for hard endpoint benefits is very limited; hyperkalemia; risks associated with concomitant use ACEI/ARB
RANI	AT1R + NEP (neprilysin/MME, zinc metalloprotease)	Simultaneously blocking AT_1_ receptors and inhibiting neutral endopeptidase (NEP), thereby increasing levels of natriuretic peptides (ANP, BNP, CNP), bradykinin, and adrenomedullin	1. Possesses all the anti-inflammatory and antioxidant effects of ARB 2. ↑Natriuretic peptide → NPR-A (particulate guanylyl cyclase) → cGMP → PKG 3. Inhibits TGF-β/Smad2/3 fibrosis signaling	★★★☆	Cardiac (core evidence for heart failure), renal, and vascular systems	Risk of hypotension, hyperkalemia, and worsening renal function; angioedema (slightly higher than ARBs), dry cough (between ACEIs and ARBs)
sMRAs	MR/NR3C2 (nuclear receptor superfamily, ligand-activated transcription factor)	Competitively blocks aldosterone binding to the MR receptor, inhibiting aldosterone-mediated genomic and non-genomic effects; spironolactone also crossbinds with androgen/progestin receptors	1. ↓NF-κB 2. ↓NLRP3 (ROS → TXNIP dissociation → NLRP3 assembly → caspase-1 → IL-1β/IL-18) 3. ↓TGF-β → TβRI/II → Smad2/3 → Smad4 → Col I/III, α-SMA, CTGF 4. ↑Nrf2 5. ↓Nox → ROS	Spironolactone ★★☆ Eplerenone ★★	Cardiac (classic evidence in heart failure), renal, and vascular systems; Primary Aldosteronism	Severe hyperkalemia; spironolactone-related hormonal adverse effects (gynecomastia, menstrual disorders), with eplerenone showing significantly lower risk than spironolactone; increased risk of eGFR decline
nsMRAs	MR/NR3C2 (highly selective, with tissue distribution biased toward heart and kidney)	Highly selective and high-affinity binding to MR receptors, without recruiting co-activators upon binding; no cross-reactivity associated with sex hormones	1. ↓NF-κB 2. ↓NLRP3 3. ↓TGF-β/Smad2/3 4. ↑Nrf2 5. ↓Nox1/Nox4→ROS 6. Clinical evidence for antifibrotic and anti-inflammatory effects is more prominent than that of sMRAs	Finerenone ★★★★	Renal (core evidence for DKD), cardiac, and vascular systems	Hyperkalemia; risk of eGFR decline; evidence for hard endpoints in DKD has been established (FIDELIO-DKD, FIGARO-DKD, please verify), while evidence for other indications is still accumulating

Evidence Grading: This table synthesizes preclinical and clinical evidence. For specific evidence levels, please refer to the corresponding sections in the main text.

Symbols:”↑” denotes upregulation/activation; “↓” denotes downreg.

Abbreviations: ACEI, angiotensin-converting enzyme inhibitor; ARB, angiotensin II receptor blocker; ARNI, angiotensin receptor-neprilysin inhibitor; MRA, mineralocorticoid receptor antagonist; sMRAs, steroidal mineralocorticoid receptor antagonists;nsMRAs, non-steroidal mineralocorticoid receptor antagonists;DRI, direct renin inhibitor; AT1R, angiotensin II type 1 receptor; AT2R, angiotensin II type 2 receptor; MR, mineralocorticoid receptor; NEP/MME, neprilysin/membrane metalloendopeptidase; Nox, NADPH oxidase; NF-κB, nuclear factor kappa-light-chain-enhancer of activated B cells; IκBα, nuclear factor of kappa light polypeptide gene enhancer in B-cells inhibitor, alpha; Nrf2, nuclear factor erythroid 2-related factor 2; Keap1, Kelch-like ECH-associated protein 1; ARE, antioxidant response element; HO-1, heme oxygenase-1; NQO1, NAD(P)H:quinone oxidoreductase 1; SOD, superoxide dismutase; GPx, glutathione peroxidase; PPARγ, peroxisome proliferator-activated receptor gamma; NLRP3, NOD-like receptor family pyrin domain containing 3; TXNIP, thioredoxin-interacting protein; TGF-β, transforming growth factor beta; Smad, small mothers against decapentaplegic; eGFR, estimated glomerular filtration rate; DKD, diabetic kidney disease; B2R, bradykinin B2 receptor; eNOS, endothelial nitric oxide synthase; sGC, soluble guanylate cyclase; NPR-A, natriuretic peptide receptor A.

## 2 Methods

### 2.1 Literature search strategy

To comprehensively identify literature regarding the modulation of inflammatory responses and oxidative stress by RAAS inhibitors, spanning from molecular mechanisms to clinical evidence, we conducted a systematic search of four major English-language databases (PubMed/MEDLINE, Web of Science, Embase, and Cochrane Library) and three Chinese databases (CNKI, Wanfang Data, and CBM). The search period was defined as 1 January 2000, to 30 June 2026. This timeframe was selected to capture the complete evolution of evidence over the past two decades, ranging from basic mechanistic studies to large-scale clinical trials.

Eligible studies were limited to peer-reviewed articles published in English or Chinese. The search strategy employed a combination of controlled vocabulary (MeSH terms and Emtree descriptors) and free-text keywords, linked using Boolean operators. Four distinct yet complementary concept modules were constructed to align with the primary objectives of this review: the clinical effects, molecular mechanisms, and causal inference (via Mendelian randomization) of RAAS inhibitors on inflammation and oxidative stress. These modules comprised: (1) RAAS Inhibitors: Including ACEIs, ARBs, DRIs, ARNIs, and MRAs; (2) Inflammatory Markers: Such as cytokines, nuclear factor kappa-B (NF-κB), C-reactive protein (CRP), and the NLRP3 inflammasome; (3) Oxidative Stress Markers: Including reactive oxygen species (ROS), NADPH oxidase, malondialdehyde (MDA), 8-hydroxydeoxyguanosine (8-OHdG), and antioxidant enzymes; (4) Mechanistic Pathways and Evidence Types: Encompassing specific signaling pathways and study designs such as randomized controlled trials (RCTs), observational studies, and meta-analyses.

### 2.2 Inclusion and exclusion criteria

Inclusion criteria included: (1) Study Design: Original research articles (including randomized controlled trials, cohort studies, case-control studies, cross-sectional studies, animal experiments, and *in vitro* cell studies), meta-analyses, and systematic reviews; (2) Intervention or Exposure: Studies explicitly evaluating the impact of RAAS inhibitors (e.g., ACEIs, ARBs, direct renin inhibitors) or key RAAS components (e.g., Angiotensin II, AT1R, AT2R) on inflammatory responses and oxidative stress; (3) Outcomes: Reporting at least one relevant outcome measure, specifically changes in inflammatory markers, oxidative stress markers, or the expression/activity of associated signaling pathway molecules, such as nuclear factor κB (NF-κB), nuclear factor erythroid 2-related factor 2/heme oxygenase-1 (Nrf2/HO-1), mitogen-activated protein kinases (MAPKs), phosphoinositide 3-kinase/protein kinase B (PI3K/Akt), adenosine monophosphate-activated protein kinase/mammalian target of rapamycin (AMPK/mTOR), and sirtuin 1 (SIRT1).

Exclusion criteria included: (1) Publication Type: Case reports, case series, narrative reviews, letters to the editor, commentaries/editorials, and conference abstracts lacking full-text availability; (2) Irrelevance to Study Objectives: Research focusing exclusively on the cardioprotective effects or other target-organ protection of RAAS inhibitors without reporting specific data or outcomes related to inflammation or oxidative stress; (3) Redundant Data: Duplicate publications involving the same cohort or experimental dataset (only the publication with the most complete data was retained); (4) Methodological Limitations: Observational studies with an insufficient sample size (n < 50) that lacked rigorous statistical analysis.

### 2.3 Literature screening and data extraction

This narrative review was conducted using a systematic search strategy. The literature screening process is depicted in a simplified flow diagram based on the PRISMA guidelines (Figure 2). Two investigators (Yin Hongtao and Yang Mengdi) independently screened all retrieved records by title and abstract according to predefined inclusion and exclusion criteria. Full texts of potentially eligible studies were subsequently retrieved and assessed for eligibility. To minimize the risk of missing key evidence, reference lists from high-impact reviews and relevant clinical guidelines were manually scrutinized to identify additional eligible studies. Final study selection and data synthesis were performed independently by two investigators, with discrepancies resolved through group discussion. Given the methodological heterogeneity of the included studies—encompassing clinical, preclinical, and mechanistic research—a single quality assessment tool was not employed for scoring; instead, the methodological characteristics of the included studies were descriptively summarized. Limitations of this review include the restriction to English- and Chinese-language publications, the absence of quantitative meta-analysis, and the potential for publication bias.

**FIGURE 2:**
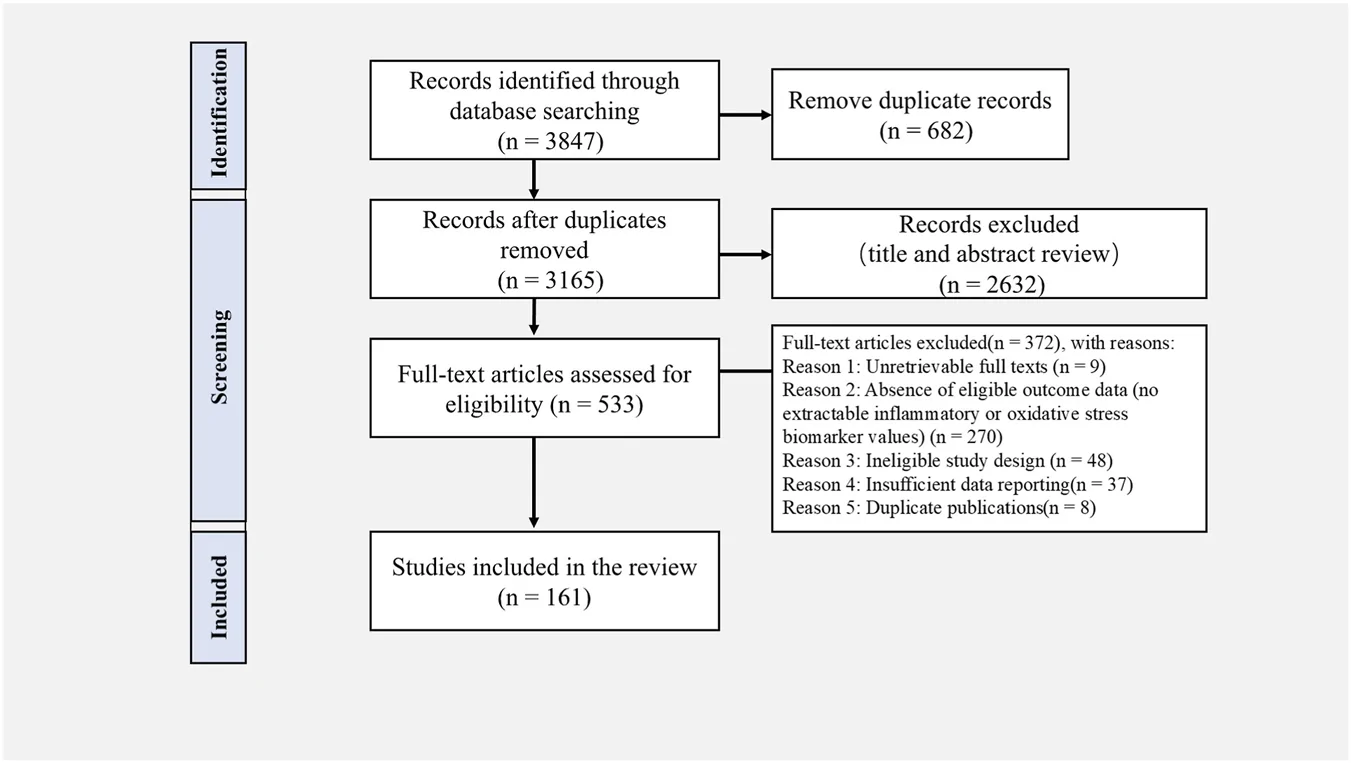
Simplified PRISMA flow diagram of study identification and selection. Records were sourced from PubMed, Web of Science, Embase, and Cochrane Library (n = 3,847). Following duplicate removal, titles and abstracts were screened (n = 3,165), and full-text articles were evaluated for eligibility (n = 533). Finally, 161 studies were included in this narrative review.

## 3 Overview of the renin-angiotensin-aldosterone system (RAAS)

The renin-angiotensin-aldosterone system (RAAS) is a complex endocrine and paracrine network, primarily consisting of renin, angiotensinogen, angiotensin-converting enzyme (ACE), angiotensin II (Ang II), and its cognate receptors. Activation of the classical RAAS pathway initiates with renin secretion from renal juxtaglomerular cells, which cleaves liver-derived angiotensinogen into angiotensin I (Ang I). Ang I is subsequently converted by ACE into Ang II—a potent vasoconstrictor with broad biological activity. Ang II exerts divergent biological effects via binding to angiotensin II type 1 receptor (AT1R) and angiotensin II type 2 receptor (AT2R); notably, AT1R mediates the majority of characterized RAAS effects, including vasoconstriction, aldosterone synthesis and secretion, sodium and water retention, myocardial hypertrophy, fibrosis, as well as inflammatory and oxidative stress responses (Ekholm and Kahan, 2021). Accordingly, the ACE/AngII/AT1R axis is implicated in a spectrum of pathological processes, such as fibrosis, inflammation, metabolic dysregulation, heart failure, cancer, aging, and diabetes mellitus.

Beyond the classical RAAS pathway, a protective RAAS axis identified in recent years has garnered increasing scholarly attention. Angiotensin-converting enzyme 2 (ACE2) not only directly and efficiently degrades angiotensin I (Ang I)—the enzymatic product of ACE—to generate the vasodilatory peptide angiotensin-(1–7) [Ang-(1–7)], but also competitively sequesters ACE’s substrate Ang I to produce angiotensin-(1–9) [Ang-(1–9)]. The latter is further hydrolyzed into Ang-(1–7) via ACE or neutral endopeptidase (NEP). Ang-(1–7) exerts a spectrum of protective effects—including vasodilation, anti-proliferation, anti-fibrosis, anti-inflammation, and antioxidant activity—through activation of the Mas receptor (Mascolo et al., 2020). The ACE2/Ang-(1–7)/Mas axis antagonizes the classical RAAS pathway, together maintaining the balance within the RAAS system. Under physiological conditions, these two pathways exist in dynamic equilibrium; however, under pathological conditions, either excessive activation of the classical pathway or relative deficiency of the protective pathway may lead to disease progression (Wan et al., 2024). Furthermore, ACE2 can convert Ang I into Ang-(1–9), which can also bind to AT2R and exert certain protective effects (Mascolo et al., 2020). This complex network regulation makes the RAAS system a multidimensional and multi-level functional network, resulting in intricate and diverse roles in modulating inflammatory responses and oxidative stress.

## 4 Mechanisms underlying the association between the RAAS system and inflammation/oxidative stress

### 4.1 Pro-inflammatory and pro-oxidative effects of the classical RAAS pathway (angiotensin II/AT1R axis)

As the central effector molecule of t the RAAS system, Ang II not only exerts potent vasoconstrictive activity but also functions as a critical pro-inflammatory and pro-oxidative mediator. In terms of its pro-inflammatory effects: First, Ang II directly promotes monocyte recruitment and adhesion to the vascular wall by upregulating the expression of adhesion molecules on the surface of endothelial cells and vascular smooth muscle cells, such as vascular cell adhesion molecule-1 (VCAM-1) and intercellular adhesion molecule-1 (ICAM-1). Second, Ang II induces the expression and secretion of multiple chemokines, including monocyte chemoattractant protein-1 (MCP-1), thereby further amplifying inflammatory cell infiltration. Third, via activation of the AT1R, Ang II induces the activation of multiple pro-inflammatory signaling cascades, including the nuclear factor κB (NF-κB) pathway, mitogen-activated protein kinase (MAPK) pathway, and transforming growth factor β (TGF-β) pathway (Ekholm and Kahan, 2021; Cantero-Navarro et al., 2021). In the NF-κB pathway, Ang II stimulates the activation of IκB kinase (IKK), which triggers the degradation of IκBα protein and the nuclear translocation of NF-κB, thereby initiating the transcription of genes encoding tumor necrosis factor-α (TNF-α), interleukin-1β (IL-1β), interleukin-6 (IL-6), and adhesion molecules. For the MAPK pathway, Ang II activates key molecules such as p38 MAPK, extracellular signal-regulated kinase (ERK), and c-Jun N-terminal kinase (JNK); these cascades collectively participate in the regulation of inflammatory responses and cellular stress responses (Yisireyili et al., 2018). Additionally, Ang II upregulates the expression of cyclooxygenase-2 (COX-2) and inducible nitric oxide synthase (iNOS), increasing the production of prostaglandin E_2_ (PGE_2_) and nitric oxide (NO), which further exacerbates inflammatory reactions.

In terms of pro-oxidative stress induction, Ang II primarily triggers oxidative stress by activating the NADPH oxidase system to increase reactive oxygen species (ROS) production (Daiber et al., 2021). In vascular endothelial cells, Ang II binding to the AT1 receptor (AT1R) upregulates the expression and activity of Nox family isoforms (including Nox1, Nox2, and Nox4) through both G protein-dependent and -independent signaling pathways (Yisireyili et al., 2018). Specifically, in the G protein-dependent pathway, AT1R couples with Gαq/11 to activate phospholipase Cβ (PLCβ), which hydrolyzes phosphatidylinositol 4,5-bisphosphate (PIP2) into second messengers such as inositol trisphosphate (IP3) and diacylglycerol (DAG). This cascade triggers endoplasmic reticulum Ca^2+^ release and Protein Kinase C (PKC) activation, thereby promoting the assembly and activation of NADPH oxidase complexes. Once activated, NADPH oxidase utilizes NADPH as an electron donor to reduce molecular oxygen to superoxide anions (O_2_^−^•), which are subsequently converted into hydrogen peroxide (H_2_O_2_) and other ROS species (Sharma et al., 2022). In a radiation-induced rat pneumonia model, radiation directly activates the ACE/AT1R pathway in immune cells and promotes ROS generation via NADPH oxidase 2 (Sharma et al., 2022). Beyond NADPH oxidase, Ang II also impairs mitochondrial respiratory chain function, leading to increased electron leakage and elevated mitochondrial ROS production. These ROS themselves act as second messengers to further activate multiple downstream inflammatory signaling pathways, including NF-κB, AP-1, MAPK pathway, and NLRP3 inflammasome, thereby forming a vicious cycle between oxidative stress and inflammatory responses (Daiber et al., 2021). This mechanism has also been validated in cardiac inflammation models: Ang II activates NADPH oxidase via AT1R, generating ROS and subsequently activating MAPK and NF-κB signaling pathways, which ultimately culminate in myocardial inflammation and fibrosis (Abareshi et al., 2017). These findings demonstrate that the Ang II/AT1R axis functions as a pro-inflammatory and pro-oxidative mediator across diverse disease states, providing the molecular basis for the protective effects of RAAS inhibitors.

### 4.2 Anti-inflammatory and antioxidant effects of protective RAAS pathways: the ACE2/Ang-(1–7)/Mas axis and ACE2/Ang-(1–9)/AT2R axis

In contrast to the classical RAAS pathway, the ACE2/Ang-(1–7)/Mas axis represents a protective RAAS pathway that counteracts the effects of Ang II in anti-inflammatory and antioxidant processes (Mascolo et al., 2020). ACE2, a carboxypeptidase, is predominantly expressed in tissues and organs, including the heart, kidneys, lungs, and vascular endothelium, with its expression levels subject to alterations under diverse pathological conditions. ACE2 catalyzes the hydrolysis of Ang II into Ang 1–7; upon binding to the Mas receptor, Ang 1–7 activates multiple signaling cascades, such as the PI3K/Akt, MAPK/ERK, and Nrf2/ARE pathways. Additionally, it modulates downstream effector molecules, including nitric oxide (NO), cyclooxygenase-2 (COX-2), and forkhead box O1 (FOXO1), thereby exerting multifaceted protective effects encompassing vasodilation, inflammation inhibition, antioxidation, proliferation suppression, and antifibrosis (Wan et al., 2024; Li et al., 2017). In a sepsis-induced cardiomyopathy (SIC) model using C57BL/6 mice, Wan TT et al. (Wan et al., 2024) demonstrated that activation of ACE2 significantly improves cardiac function while attenuating inflammatory responses and oxidative stress, which is mediated by the promotion of mitochondrial biogenesis via the Mas receptor-Sirt1 pathway. Further investigations confirmed the protective role of the ACE2/Ang-(1–7)/Mas axis, as the ACE2 inhibitor MLN-4760 and Mas antagonist A779 were shown to abrogate most of these protective effects (Wan et al., 2024; Sampaio et al., 1979). Additionally, the protective effect of Ang-(1–7) was absent in Mas-deficient animals (Sampaio et al., 1979; Alenina et al., 2008; Santos et al., 2018). However, in contrast to the above findings, the Ang-(1–7)/Mas receptor axis may also promote inflammation under certain conditions. Previous studies have reported that systemic administration of Ang-(1–7) induces renal inflammation in mice by activating nuclear factor-κB (NF-κB) and related pro-inflammatory genes (Esteban et al., 2009). Moreover, studies using Mas receptor (MasR)-deficient mice or AT1R/AT2R double-knockout mice revealed that Ang-(1–7)-induced renal inflammatory responses are entirely mediated by MasR (Esteban et al., 2009). This inconsistent effect of the Ang-(1–7)/Mas receptor axis may be attributed to the complexity of Ang-(1–7) signaling, as Ang-(1–7) can also activate Mas-related G protein-coupled receptor D (MrgD)—a receptor for the angiotensin-derived peptide alamandine (Tetzner et al., 1979). Nevertheless, prevailing evidence suggests that the Mas receptor-mediated PI3K/Akt-eNOS pathway constitutes a core mechanism of endogenous anti-inflammatory and antioxidant protection. This pathway exerts regulatory “braking” effects via NO-dependent suppression of inflammasome activation. Consequently, these findings provide a theoretical rationale for the development of novel therapeutic strategies based on ACE2 activation or Ang-(1–7) supplementation.

In addition, as another critical component of the protective arm of the RAAS system, the AT2R is also recognized to possess anti-inflammatory and antioxidant potential (Cantero-Navarro et al., 2021). While research on AT2R is less extensive than that on the AT1R, AT2R has been acknowledged for its role in counteracting and modulating AT1R-mediated signaling (Valentini et al., 2025). Recent studies have demonstrated that the binding of Ang II to AT2R activates multiple phosphatases, including mitogen-activated protein kinase phosphatase-1 (MKP-1) and Src homology region 2 domain-containing phosphatase-1 (SHP-1). It also enhances bradykinin expression, reduces NAD(P)H oxidase activity, reactive oxygen species (ROS) formation, and inflammatory cytokine synthesis, thereby exerting anti-inflammatory, antioxidant, and cardioprotective effects (Mascolo et al., 2020; Valentini et al., 2025; Roca-Ho et al., 2020). Additionally, other studies have indicated that AT2R exhibits neuroprotective properties, which may be linked to neural regeneration and defense against oxidative stress (Qi et al., 2011). However, the precise role of AT2R in regulating inflammation and oxidative stress remains inconclusive based on current evidence, necessitating further in-depth investigations to clarify (Cantero-Navarro et al., 2021).

### 4.3 Cross-talk between the RAAS system and other inflammatory and oxidative stress signaling pathways

Research has demonstrated that the RAAS system engages in an intricate cross-talk network with other inflammatory and oxidative stress signaling pathways, endowing RAAS inhibitors with multi-targeted and multi-pathway biological effects. Nuclear factor erythroid 2-related factor 2 (Nrf2), a pivotal transcription factor governing the cellular antioxidant defense system, exerts a central regulatory role in inflammatory responses and oxidative stress (Hu et al., 2022). In a rat model of diabetic cardiomyopathy (DCM) established via high-fat diet feeding combined with intraperitoneal streptozotocin injection, the protective effects of the ACE2/Ang 1–7/Mas axis were shown to be partially dependent on Nrf2 pathway activation. Aliskiren, by blocking Ang II receptors, modulates the SIRT1/Nrf2/NF-κB signaling pathway through the ACE2/Ang 1–7/Mas axis to mitigate diabetes-induced oxidative stress and inflammation (Jin et al., 2021). Similarly, studies investigating lisinopril’s protective effects against cisplatin-induced testicular injury have confirmed the critical involvement of the Nrf2/Keap1/HO-1 pathway in the antioxidant and anti-inflammatory actions of ACE inhibitors (Arafa et al., 2025).

A recent study has demonstrated that the Janus kinase-signal transducer and activator of transcription (JAK-STAT) signaling pathway may also serve as one of the critical pathways mediating the pro-inflammatory responses of the RAAS system. In an adjuvant-induced arthritis model in rats, Arab HH et al. (Arab et al., 2022) observed that lisinopril mitigated adjuvant-induced joint inflammation and cartilage degradation by suppressing the JAK-2/STAT-3/receptor activator of nuclear factor-κB ligand (RANKL) axis. Concurrently, lisinopril upregulated the Nrf2/HO-1 antioxidant pathway, elevated glutathione (GSH) levels and superoxide dismutase (SOD) activity, and reduced the levels of lipid peroxidation products. In addition, peroxisome proliferator-activated receptor gamma (PPARγ), a ligand-activated transcription factor, has been implicated in the pro-inflammatory responses mediated by the RAAS system. In a study investigating lipopolysaccharide (LPS)-induced injury in human renal tubular epithelial HK-2 cells, Zeng et al. (Zeng et al., 2026) demonstrated that ganoderic acid A exerts protective effects via the PPARγ/NF-κB pathway, leading to the hypothesis that PPARγ agonists may attenuate inflammatory responses by suppressing the NF-κB signaling pathway. While direct evidence linking RAAS inhibitors to the PPARγ pathway remains limited, modulation of PPARγ expression by ACEI and ARB has been documented (Singh et al., 2024), suggesting this may represent an additional mechanism underlying the anti-inflammatory actions of RAAS inhibitors.

These observations indicate that the mechanistic complexity of the RAAS-inflammation-oxidative stress network substantially surpasses the simplified premises underlying current clinical intervention strategies. At the molecular level, the dynamic equilibrium between the AT1R-Nox2-NF-κB/MAPK axis and the ACE2/Ang-(1–7)/Mas axis, coupled with the reciprocal regulation of RAAS components by inflammatory mediators and the interplay between mitochondrial dysfunction and antioxidant defense exhaustion, collectively constitute a highly intricate, dynamic network characterized by multiple positive and negative feedback loops (Sahin and Ergul, 2022). Furthermore, cross-disease evidence underscores that the vicious cycle of inflammation and oxidative stress represents a pervasive pathophysiological mechanism. As a pivotal regulatory node within this cycle, the RAAS warrants deeper investigation regarding its therapeutic potential.

### 4.4 Molecular mechanisms underlying the anti-inflammatory and antioxidant effects of different classes of RAAS inhibitors: direct receptor blockade versus downstream second messenger activation

The molecular mechanisms underlying the anti-inflammatory and antioxidant effects of RAAS inhibitors can be categorized into two distinct classes based on their sites of action: direct receptor blockade and modulation of downstream second messenger signaling. ARBs and MRAs exert their primary effects through direct inhibition of AT1R and MR, respectively. As a G protein-coupled receptor (GPCR), AT1R activation initiates multiple downstream signaling cascades—including Gq/Ca^2+^, cAMP/PKA, ROS, phosphatidic acid (PA), and cGMP pathways—that collectively regulate cellular phenotypes (Nishiguchi et al., 2020). Notably, significant heterogeneity exists in the off-target profiles among MRAs. Spironolactone exhibits cross-reactivity with androgen and progesterone receptors, resulting in hormone-related adverse effects. In contrast, finerenone demonstrates high selectivity for MR without recruiting coactivators, thereby minimizing hormonal cross-interference. Furthermore, the molecular characteristics of ARBs are not uniform; while telmisartan possesses partial PPARγ agonist activity alongside its AT1R-blocking function—enabling suppression of NF-κB and AP-1 transcriptional activity via PPARγ—evidence for similar PPARγ-mediated effects in other ARBs remains inconsistent. This structural and functional divergence suggests that the pleiotropic anti-inflammatory and antioxidant benefits observed within the ARB class cannot be attributed to a homogeneous class effect.

Enzyme-level inhibition is exemplified by ACEIs, DRIs, and ARNIs. ACEIs attenuate Ang II synthesis and elevate bradykinin levels via ACE inhibition; DRIs reduce Ang I generation through renin suppression; and ARNI combines neprilysin inhibition to potentiate the natriuretic peptide system with AT1 receptor blockade. All three mechanisms involve remodeling of downstream second messenger networks (Oatmen et al., 2018). Critically, these agents do not directly activate second messengers; rather, their modulation of cyclic guanosine monophosphate (cGMP) and nitric oxide (NO) pathways occurs indirectly via alterations in upstream ligand concentrations. Specifically, ACEIs increase cGMP through the bradykinin/B2R/eNOS/NO/sGC axis, whereas ARNIs elevate cGMP via the natriuretic peptide/NPR-A/particulate guanylyl cyclase axis. This indirect mode of action fundamentally distinguishes enzyme inhibition from direct second messenger regulation. Consequently, receptor blockade and downstream network modulation are not mutually exclusive paradigms but represent complementary ends of the RAAS therapeutic spectrum. The net pharmacological efficacy of RAAS inhibitors is determined by their specific positioning along two dimensions: the hierarchical level of intervention and target selectivity profile, rather than by inherent class-specific attributes alone.

## 5 Anti-inflammatory and antioxidant effects of different classes of RAAS inhibitors

### 5.1 Angiotensin-converting enzyme inhibitors (ACEIs)

Angiotensin-converting enzyme inhibitors (ACEIs) constitute the prototypical class of renin-angiotensin-aldosterone system (RAAS) modulators and were among the first agents scrutinized for their capacity to mitigate inflammation and oxidative stress. Mechanistically, ACEIs suppress angiotensin II (Ang II) biosynthesis via ACE inhibition while concurrently impeding bradykinin degradation. Given the intrinsic vasodilatory, antiproliferative, and anti-inflammatory attributes of bradykinin, the therapeutic efficacy of ACEIs may be partially mediated by enhanced bradykinin bioavailability (Kostakoglu et al., 2020). Furthermore, in contrast to ARBs, ACEIs potentiate ACE2 activity, thereby facilitating the generation of Ang-(1–7), which exerts counter-regulatory effects against Ang II-mediated pathology (Mirabito Colafella and Danser, 1979). A robust body of preclinical and clinical evidence corroborates that ACEIs confer substantial anti-inflammatory and antioxidant benefits across a spectrum of disease models.

Recent studies utilizing *in vitro* assays and animal models have substantiated that various ACEIs, including captopril (Armstrong et al., 2026; Abareshi et al., 2017; Sahin and Ergul, 2022; Azizi-Malekabadi et al., 2020; Boskabadi et al., 2018; Boskabadi et al., 2019; Al-Hashem et al., 2021; El-Ashmawy et al., 2018; Mostafa et al., 2025), benazepril (Liu et al., 2011; Liu et al., 2009; Zhan et al., 2021), lisinopril (Sharma et al., 2022; Arafa et al., 2025), perindopril (Kostakoglu et al., 2020; Barhoumi et al., 2021; Pastacı Özsobacı et al., 2024), enalapril (Zhou et al., 2015; Kim et al., 2011) and ramipril (Yadin et al., 2023; Mazumdar and Biswas, 2025), exert significant protective effects across a spectrum of pathological conditions, such as cardiac fibrosis, heart failure, renal insufficiency, pulmonary inflammation, sepsis, hepatotoxicity, acute pancreatitis, and neuroinflammation. Mechanistically, these agents markedly downregulate the expression of pro-inflammatory cytokines—including TNF-α, IL-6, IL-1β, and CRP—by inhibiting the Ang II/NF-κB/TGF-β axis, while concurrently modulating oxidative stress biomarkers such as MDA, SOD, and ROS. Specifically, in a doxorubicin (DOX)-induced injury model using rat embryonic cardiomyocytes (H9c2 cells), Zhan et al. (Zhan et al., 2021) demonstrated that benazepril pretreatment attenuates DOX-induced oxidative stress and suppresses apoptosis via the PI3K/Akt signaling pathway. Furthermore, investigations in human cardiomyocytes (AC16 cells) revealed that lisinopril confers protection against oxidative stress and fibrogenesis through the activation of Sirtuin 1 (SIRT1) and Sirtuin 6 (SIRT6) pathways (Scisciola et al., 2022).

Furthermore, in a rat model of radiation-induced pneumonitis, Sharma et al. demonstrated that radiation-triggered ACE activation promotes pulmonary interstitial inflammation via immune modulation. Specifically, the ACE inhibitor lisinopril attenuates the activation of pro-inflammatory immune cell subsets. Subsequent *in vitro* experiments confirmed that lisinopril abrogates radiation-induced reactive oxygen species (ROS) generation by inhibiting NADPH oxidase 2 or blocking the AT1 receptor. These findings implicate the Ang II/AT1R/NADPH oxidase axis as a pivotal mediator of radiation-induced oxidative stress and highlight the therapeutic role of ACEIs in disrupting this pathological cascade (Sharma et al., 2022). In a complementary study focusing on inflammatory balance, Boskabadi et al. assessed markers in bronchoalveolar lavage fluid (BALF) following LPS-induced lung injury. They reported that captopril significantly reduced levels of malondialdehyde (MDA), IFN-γ, PGE2, and TGF-β1, while concurrently elevating total thiols, SOD activity, and IL-4. Notably, captopril dose-dependently restored the homeostasis between pro- and anti-inflammatory cytokines, thereby correcting the Th1/Th2 imbalance. This suggests that beyond simple antioxidant effects, ACEIs exert specific immunomodulatory actions to resolve acute inflammatory responses (Boskabadi et al., 2019). The mechanistic breadth of ACEIs is further evidenced in bone metabolism research. While previous studies emphasized the inhibition of the classical ACE/AngII pathway, recent work by Liu et al. (Liu et al., 2022) highlights the involvement of the protective non-classical ACE2/Ang-(1–7)/Mas receptor pathway. In a glucocorticoid-induced osteonecrosis of the femoral head (GIONFH) rat model, captopril reversed methylprednisolone-induced upregulation of ACE1, AT1 receptor, and RANKL, while restoring the expression of ACE2, Mas receptor, and osteoprotegerin. *In vitro* validation confirmed that captopril mitigates dexamethasone-induced apoptosis and oxidative stress by activating the ACE2/Mas signaling axis, thereby promoting osteogenic recovery. These findings not only confirm the anti-inflammatory and antioxidant properties of ACEIs but also elucidate their potential as multi-target agents in orthopedic therapies (Liu et al., 2022).

Clinical studies have provided further evidence for the pleiotropic effects of ACE inhibitors (ACEIs) beyond blood pressure control. A small-sample observational study in Chinese pediatric patients with IgA nephropathy demonstrated that early initiation of ACEI therapy significantly attenuated systemic oxidative stress, evidenced by reduced plasma levels of advanced oxidation protein products (AOPP) and malondialdehyde (MDA), alongside elevated superoxide dismutase (SOD) activity (Pei et al., 2016). In a more recent investigation involving patients with concurrent Alzheimer’s disease and hypertension, Qaisar et al. (Qaisar et al., 2026) reported that 12 months of perindopril treatment significantly lowered plasma concentrations of inflammatory markers (CRP, IL-6) and an oxidative stress biomarker (8-isoprostane). Notably, these improvements in functional status appeared to be independent of the drug’s antihypertensive efficacy, suggesting a direct modulation of neuroinflammation and oxidative damage. Furthermore, a multicenter study involving 3,044 Chinese patients with concurrent hypertension and intracranial aneurysms demonstrated that RAAS inhibitor use was significantly associated with a reduced risk of aneurysm rupture (OR = 0.490), likely mediated by RAAS pathway inhibition and its subsequent antioxidant and anti-inflammatory effects (Zhong et al., 1979). In the context of cardioprotection targeting oxidative stress and inflammation, large-scale trials have shown that cytokine-targeted monoclonal antibodies reduce cardiovascular mortality in patients with established atherosclerosis. Similarly, ACEIs exhibit pleiotropic immunomodulatory benefits, with the normalization of oxidative stress emerging as a common mechanistic feature across these therapies (Daiber et al., 2021). Moreover, a recent meta-analysis of 32 randomized controlled trials (n = 3,489) indicated that while ACEIs significantly lowered CRP, IL-6, and TNF-α levels, ARBs only showed consistent efficacy in reducing IL-6 (Awad et al., 2022). Collectively, these findings suggest that the systemic anti-inflammatory and antioxidant properties of ACE inhibitors confer broad therapeutic benefits across diverse pathologies.

However, the alignment between hard clinical outcomes and biomarker-based inflammation metrics is discordant, indicating a critical disconnect in translational medicine. For example, despite the robust antihypertensive effects of ROS scavengers in preclinical models, clinical evidence for antioxidant therapy remains equivocal, suggesting substantial hurdles between mechanistic proof-of-concept and clinical endpoint achievement. Among radiotherapy-treated cancer patients, ACEIs significantly reduced the incidence of grade ≥2 radiation pneumonitis (OR: 0.50; 95% CI: 0.32–0.77), whereas their effect on survival varied heterogeneously by tumor type and stage (Liao et al., 2025). Consequently, although ACEI-mediated anti-inflammatory and antioxidant activities are detectable via biomarkers, their quantitative contribution to hard endpoints, such as MACE reduction, has not been fully established. Furthermore, the protective role of ACEIs is not uniform; conversely, in Crohn’s disease cohorts, ACEI exposure has been paradoxically associated with adverse long-term prognoses (Karime et al., 2024), which contradicts the protective effects observed in preclinical studies. This inconsistency may stem from differences in the pathogenesis of distinct diseases, or from the differential impacts of ACEI-mediated inhibition of intestinal ACE on local RAAS system activity and the metabolism of bradykinin and substance P (Salmenkari et al., 2018). Further investigation indicates that within the intestinal microenvironment, the net physiological effects of bradykinin and substance P are predominantly pro-inflammatory, pro-secretory, and pro-edematous, contrasting sharply with their vasodilatory, anti-inflammatory, and anti-remodeling roles observed in the systemic vascular bed (Landry et al., 2023). Inhibition of intestinal ACE by ACEIs simultaneously impairs the degradation of both bradykinin and substance P, leading to their accumulation in the intestinal mucosa. This accumulation activates specific receptors—namely, the B1/B2 bradykinin receptors and the neurokinin 1 receptor (NK1R)—thereby triggering a synergistic cascade of neurogenic inflammation and immune cell activation that ultimately drives intestinal mucosal inflammation. Central to this mechanism is the dual role of ACE as a common degradative enzyme for both neuropeptides; thus, ACEI-induced blockade affects not only bradykinin metabolism but also concurrently inhibits substance P catabolism (Choi et al., 2023; Davin et al., 2019). Similarly, no anti-inflammatory protective effects of ACEIs were observed in several other studies, including those involving maintenance hemodialysis (MHD) patients (Kaseda et al., 2018), oral cancer patients (Du et al., 2020), and hypertensive patients with age-related macular degeneration (Subramanian et al., 2022). Thus, it is inappropriate to simply generalize the findings of ACEIs in one disease to other disease contexts (Karime et al., 2024; Salmenkari et al., 2018). The underlying basis for this tissue-specific discrepancy resides in the heterogeneity of tissue microenvironments, receptor expression profiles, and downstream signaling cascades.

Accordingly, a cross-scale evidence base has emerged supporting the anti-inflammatory and antioxidant-stress properties of ACEIs, with preclinical findings showing considerable consistency. However, it is important to note that while these effects are detectable at the biomarker level, their quantitative association with reduced MACE risk remains insufficiently elucidated. Furthermore, the primary evidence for these mechanisms largely derives from basic experimental studies and small-scale clinical trials, or from indirect observations in patients with hypertension and cardiovascular disease. Most clinical trials have not designated inflammatory or oxidative stress markers as primary endpoints, and meta-analyses have failed to systematically isolate the independent effects of ACEIs on circulating biomarkers (Habib et al., 2025; Daiber et al., 2021). Consequently, further rigorous clinical research is warranted to validate the translational applicability of these findings to human pathological conditions.

### 5.2 Angiotensin II receptor blockers (ARBs)

Angiotensin II receptor blockers (ARBs) exert their therapeutic effects through selective blockade of the AT1 receptor. Unlike ACE inhibitors, ARBs do not interfere with bradykinin metabolism, resulting in a lower incidence of adverse effects and establishing them as the preferred class of RAAS inhibitors for the management of hypertension. Recent extensive preclinical research involving cell cultures and animal models has demonstrated that various ARBs—including losartan (Chen et al., 2022; Xu et al., 2021; Qin et al., 2018; Karanovic et al., 2016), valsartan (Cheng et al., 2020; Kutbi and Almalki, 2024; Jiao et al., 2011; Zhou et al., 2014), irbesartan (Yisireyili et al., 2018; Helal and Samra, 2020; Al-Kuraishy et al., 2020), telmisartan (Singh et al., 2024; Lakshmanan et al., 2011; Rodríguez-Lara et al., 2019; Arab et al., 2014; Pang et al., 2012), candesartan (Sueta et al., 2012; Atwa et al., 2022), olmesartan (Abo El-Nasr et al., 2022; Aziz et al., 2020) and azilsartan (Jin et al., 2021; Pan et al., 2021)—effectively attenuate angiotensin II-mediated pro-inflammatory and pro-oxidative responses via AT1R antagonism. The underlying mechanisms involve the inhibition of NAD(P)H oxidase activity, downregulation of NF-κB and MAPK signaling pathways, reduction of reactive oxygen species (ROS) generation, and restoration of antioxidant enzyme function. Furthermore, certain ARBs exhibit unique pleiotropic properties, such as PPAR-γ/PPAR-α agonism and enhancement of the protective ACE2/Ang-(1–7)/Mas axis (Singh et al., 2024; Nakano et al., 2016).

Losartan is one of the most extensively characterized ARBs, with its anti-inflammatory and antioxidant properties firmly established through numerous *in vitro* and *in vivo* studies. Recent evidence indicates that losartan attenuates sepsis-induced cardiomyopathy (SIC) by modulating macrophage polarization via TLR4-mediated NF-κB and MAPK signaling pathways, reducing oxidative stress, and inhibiting the infiltration of neutrophils, monocytes, and macrophages into the myocardium and spleen (Chen et al., 2022). Similarly, Jin et al. (Jin et al., 2021) demonstrated that azilsartan mitigates hyperglycemia-induced oxidative stress and inflammation in a rat model of diabetic cardiomyopathy by regulating the SIRT1/Nrf2/NF-κB signaling axis, thereby reducing collagen deposition and cardiomyocyte apoptosis. Furthermore, in both doxorubicin-induced cardiomyopathy models in male rats and adriamycin-induced myocardial injury models, azilsartan (Al-Chlaihawi and Janabi, 2023) and valsartan (Cheng et al., 2020) were found to alleviate cardiotoxicity. These agents suppressed cardiac levels of TNF-α, IL-1β, malondialdehyde (MDA), and Caspase-3, while concurrently upregulating total antioxidant capacity (TAC) and Bcl-2 expression, collectively attenuating apoptosis, inflammation, and oxidative stress within cardiac tissues.

Notably, certain ARBs, such as telmisartan, exert additional anti-inflammatory effects through peroxisome proliferator-activated receptor-gamma (PPARγ) activation. Telmisartan, candesartan, and irbesartan have been characterized as ARBs with partial PPARγ agonist activity, mediating metabolic regulation and inflammation suppression. Previous research demonstrated that telmisartan attenuates oxidative stress and renal fibrosis in metformin-induced diabetic mice by modulating Angiotensin-(1–7)/Mas receptor expression, concurrently exhibiting PPARγ agonist-like properties (Lakshmanan et al., 2011). In the context of pancreatic inflammatory microenvironments, telmisartan also confers distinct advantages via PPARγ-dependent anti-inflammatory mechanisms (Singh et al., 2024). Regarding immunomodulation, while ACEIs demonstrate therapeutic potential in autoimmune myocarditis, colitis, and diabetic nephropathy primarily through cytokine attenuation, the immunomodulatory efficacy of ARBs is more closely linked to PPARγ activation pathways (Haliga et al., 2025). Furthermore, in rat models of lung injury, candesartan has been shown to prevent cisplatin (CIS)-induced pulmonary damage by inhibiting TLR-4/NF-κB and JAK1/STAT3 signaling cascades, while upregulating PPARγ and Nrf2/HO-1 pathways to mitigate oxidative stress (Atwa et al., 2022). Collectively, these findings indicate that specific ARBs—particularly telmisartan and candesartan—provide supplementary anti-inflammatory benefits via PPARγ activation (Haliga et al., 2025).

In addition, in hepatic injury models, irbesartan attenuated acetaminophen (APAP)-induced liver inflammation by downregulating the expression of NF-κB, phosphorylated NF-κB, and TNF-α. It also mitigated hepatotoxicity, oxidative stress, and apoptosis through a reduction in the Bax/Bcl-2 ratio and suppression of caspase-3 expression and activation (Helal and Samra, 2020). Similarly, in nephrotoxicity models, methotrexate (MTX) administration elevated serum renal and inflammatory biomarkers while depleting antioxidant reserves (SOD and GSH) and increasing lipid peroxidation. Valsartan was shown to attenuate MTX-induced acute kidney injury in rats by inhibiting renal inflammation and oxidative stress, thereby highlighting its renoprotective potential (Kutbi and Almalki, 2024). Furthermore, studies have shown that valsartan can also slow the progression of diabetic nephropathy in db/db mice by reducing podocyte injury, renal oxidative stress, and inflammation (Zhou et al., 2014). Notably, the mechanisms by which ARBs regulate inflammation and oxidative stress have been further validated in bone metabolism. In an ovariectomy-induced osteoporosis mouse model, azilsartan was found to inhibit receptor activator of nuclear factor kappa-B ligand (RANKL)-induced reactive oxygen species (ROS) production and activate the Nrf2 signaling pathway by suppressing the MAPK/NF-κB signaling cascade. These effects collectively inhibit osteoclast differentiation and bone resorption, thereby ameliorating bone loss in ovariectomized mice (Pan et al., 2021).

Clinical evidence supports these mechanistic observations, although derived primarily from small-scale or retrospective studies. A randomized multicenter trial in South Korea demonstrated that valsartan and amlodipine attenuated oxidative stress biomarkers in patients with type 2 diabetes and hypertension (Kim HJ. et al., 2017). Similarly, Mostafa et al. reported that candesartan significantly reduced serum IL-1β, TNF-α, C-reactive protein (CRP), and erythrocyte sedimentation rate (ESR) in rheumatoid arthritis patients, effects comparable to those of atorvastatin (Mostafa et al., 2020). This suggests ARBs possess anti-inflammatory and immunomodulatory properties in this context, though their antioxidant efficacy may be inferior to statins. Furthermore, a retrospective analysis of the Lean European Open Survey on SARS-CoV-2 (LEOSS) registry (n = 1,946 patients with cardiovascular comorbidities or hypertension) indicated that ARB use was associated with reduced disease severity, potentially mediated by attenuated systemic inflammation (Cremer et al., 2021). Corroborating this, a recent randomized, double-blind, placebo-controlled pilot trial in outpatients with SARS-CoV-2 infection demonstrated that telmisartan activated the protective ACE2/Ang-(1–7)/Mas axis within the RAAS, thereby initiating anti-inflammatory cascades and mitigating systemic inflammation (Lu et al., 2025). However, analogous to ACEIs, the evidence for ARBs reveals an inconsistency between improvements in inflammatory biomarkers and hard clinical outcomes, highlighting a significant translational disconnect. Although the anti-inflammatory and antioxidant effects of ARBs are detectable at the biomarker level, their quantitative association with hard endpoint benefits, such as MACE reduction, remains incompletely elucidated.

Furthermore, clinical evidence regarding the biological effects of ARBs remains inconsistent. Baker et al. (Baker et al., 2021) reported that losartan failed to improve systemic inflammatory markers or facilitate T-cell immune reconstitution in older patients with perinatally acquired HIV (PHIV) who had achieved viral suppression. Similarly, the SAFIR study—a randomized, double-blind, placebo-controlled trial evaluating the long-term impact of irbesartan on inflammatory biomarkers in maintenance hemodialysis patients—observed no significant acute or sustained reduction in these markers, suggesting that ARB therapy may not provide anti-inflammatory protection in this specific population (Peters et al., 2017). However, these null findings should be interpreted with caution due to subgroup heterogeneity, variations in study design, and distinct demographic characteristics. Consequently, they cannot be broadly generalized; indeed, the prevailing body of evidence continues to support the protective roles of ARBs against inflammation and oxidative stress across various disease models.

Collectively, preclinical evidence substantiates that ARBs modulate inflammation and oxidative stress via multifaceted mechanisms. These effects are consistently validated in models of atherosclerosis, myocardial remodeling, hypertensive target organ damage, and various renal diseases, extending to broader domains including neurodegeneration, autoimmune disorders, tumor microenvironments, and bone metabolism. However, analogous to ACE inhibitors, there is notable discordance between hard clinical outcomes and improvements in inflammatory biomarkers, highlighting a significant 'translational gap.’ Current evidence for the anti-inflammatory and antioxidant properties of ARBs remains predominantly derived from basic research and indirect observations in patients with hypertension, cardiovascular, or pulmonary conditions. Most clinical data originate from observational studies or post-hoc analyses, while prospective randomized controlled trials employing inflammatory or oxidative biomarkers as predefined primary endpoints remain scarce (Habib et al., 2025).

### 5.3 Direct renin inhibitors (DRIs)

Direct renin inhibitors (DRIs) attenuate the generation of angiotensin I (Ang I) and angiotensin II (Ang II) by directly inhibiting renin activity, thereby blocking RAAS activation at its origin. From a mechanistic perspective, given that renin serves as the rate-limiting enzyme in the RAAS cascade, its inhibition theoretically provides upstream blockade, potentially conferring pleiotropic benefits—including anti-inflammatory, antioxidant, and antifibrotic effects—beyond blood pressure reduction. These hypotheses have been substantiated by extensive preclinical animal studies (Thomas et al., 2013; Zhao et al., 2021; Yang et al., 2025). However, human clinical trials have yielded contrasting findings (Massolini et al., 2020; Pantzaris et al., 2017).

In preclinical animal studies, Thomas CM et al. (Thomas et al., 2013) demonstrated that aliskiren significantly attenuated cardiac oxidative stress and circulating inflammatory cytokines in adult diabetic mice, conferring cardioprotective effects comparable to those of ARBs and ACEIs. Consistent with these observations, Zhao Z et al. (Zhao et al., 2021) found that aliskiren may exert cardioprotective effects in rapid atrial pacing (RAP)-induced atrial remodeling in dogs, with its mechanism potentially involving anti-inflammatory activity, suppression of oxidative stress, and modulation of the PI3K/Akt signaling pathway. A recent study indicated that aliskiren may protect against hypertensive renal injury by regulating the balance between the ACE-Ang II-AT1R axis and the ACE2-Ang-(1–7)-MasR axis, as well as by inhibiting oxidative stress (Yang et al., 2025). Additionally, aliskiren’s anti-inflammatory and antioxidant protective effects have been validated in rat models of diabetic nephropathy (DN) (Mahfoz et al., 2016), diabetic hippocampal cognitive impairment (Youssef et al., 2020), arthritis (Azouz et al., 2020), diabetic liver injury (Mahfoz and Gawish, 2022), ovarian ischemia-reperfusion injury (Bayir et al., 2016), and pleurisy (Bayir et al., 2019). However, although aliskiren exhibits blood pressure-independent anti-inflammatory and antioxidant potential in preclinical studies, these findings cannot be directly extrapolated to clinical efficacy in humans. This caution is warranted given the well-documented interspecies variations in RAAS component expression, renin substrate specificity, and chymase-mediated angiotensin II generation.

A small-scale clinical trial demonstrated that aliskiren attenuated oxidative stress in patients with non-diabetic chronic kidney disease (NDCKD) and potentially improved tubular function, with no significant difference observed compared to enalapril (Renke et al., 2014), however, the evidence strength remains limited due to the small sample size. Conversely, a study investigating the long-term effects of aliskiren on vascular function in hypertensive diabetic patients revealed inconsistent findings: although aliskiren significantly upregulated the expression of eNOS phosphorylated at Ser-1177 in endothelial cells, both aliskiren and ramipril exerted negligible effects on systemic oxidative stress biomarkers. However, it is crucial to acknowledge that proof-of-concept studies supporting the use of DRIs in diabetic nephropathy are exceedingly scarce. Among clinical trials utilizing plasma renin activity (PRA) as an outcome measure, only three have been conducted. Although five DRI-related studies encompassed 10,393 patients, PRA was measured in merely 370 individuals (3.6%). Furthermore, key trials such as ALTITUDE and ASTRONAUT reported neutral or negative results for major cardiovascular and renal hard endpoints (Massolini et al., 2020; Pantzaris et al., 2017). Conversely, significant safety concerns have been raised regarding the combination of DRIs with ACEIs/ARBs, which is associated with an increased risk of hyperkalemia, hypotension, and worsening renal function (Alshahrani, 2020; Alshahrani, 2023). Pathophysiologically, the failure of combination therapy can be attributed to the disruption of compensatory mechanisms resulting from excessive RAAS suppression, while the synergistic effects of electrolyte imbalances and hemodynamic instability further exacerbate safety risks (Alshahrani, 2023). Consequently, such combination regimens should not be employed without rigorous monitoring. Future research directions should prioritize refined patient stratification and the identification of potential responder subgroups who may derive specific benefits from DRI therapy. In conclusion, human clinical evidence regarding the anti-inflammatory and antioxidant effects of DRIs remains limited and largely derived from small-scale studies, necessitating further investigation to validate these findings.

### 5.4 Mineralocorticoid receptor antagonists (MRAs)

Chronic sustained activation of the RAAS system and excessive aldosterone secretion can induce inflammatory responses and fibrosis in myocardial, vascular, and renal tissues, ultimately leading to structural and functional dysfunction. Studies have demonstrated that these detrimental effects are primarily mediated via genomic and non-genomic mineralocorticoid receptor (MR) pathways. Both steroidal and non-steroidal mineralocorticoid receptor antagonists (s-MRAs and ns-MRAs) can partially or fully block the aforementioned signaling cascades, thereby exerting aldosterone-antagonizing protective effects in heart failure and chronic kidney disease (CKD) (Fioretti et al., 2025).

In a study utilizing a sepsis rat model (Al-Kadi et al., 2022), three RAAS inhibitors—ramipril, losartan, and spironolactone—exerted protective effects, as evidenced by improved 24-h survival rates (80% in the ramipril and spironolactone groups, 60% in the losartan group, versus only 10% in the sepsis control group), enhanced renal function parameters, and reduced levels of oxidative stress and inflammatory markers. This study provides critical experimental evidence supporting the potential therapeutic value of RAAS inhibitors in sepsis. A recent study demonstrated that spironolactone pretreatment in an osteoblast oxidative damage model attenuated 2-deoxy-D-ribose (dRib)-induced elevations in cytokine levels, reactive oxygen species (ROS) production, mitochondrial oxidative load, and endoplasmic reticulum (ER) stress. Additionally, it preserved mitochondrial function and augmented antioxidant defense by increasing glutathione (GSH) levels and glyoxalase I activity, highlighting the potential utility of spironolactone in managing diabetes-related bone fragility (Park et al., 2026). Consistent with these observations, Peter MU et al. (Peter et al., 2022) found that low-dose spironolactone mitigated cardiovascular and renal complications in a polycystic ovary syndrome (PCOS) rat model by reducing oxidative stress and the expression of BAX and inflammasomes.

In clinical practice, spironolactone and eplerenone are widely used mineralocorticoid receptor antagonists (s-MRAs), often combined with renin-angiotensin system (RAS) blockers as therapeutics for heart failure and anti-proteinuric agents. Spironolactone has been demonstrated to reduce levels of inflammatory markers (including CRP and IL-6) in patients with chronic heart failure, yet it is associated with a higher risk of hyperkalemia (Zhang et al., 2025; Lin et al., 2021). However, three recent pivotal phase 3 clinical trials—FIDELIO-DKD (Bakris et al., 2020), FIGARO-DKD (Pitt et al., 2021), and their pooled analysis FIDELITY (Agarwal et al., 2022)—have unequivocally shown that finerenone, a non-steroidal mineralocorticoid receptor antagonist (ns-MRA), can decrease proteinuria, the risk of chronic kidney disease (CKD) progression, and cardiovascular (CV) risk in patients with type 2 diabetes mellitus (T2DM) and CKD by specifically inhibiting inflammation and fibrosis, while exhibiting a lower hyperkalemia risk (González-Juanatey et al., 2023). A small-sample clinical study conducted in China corroborated these findings. In a randomized controlled trial involving 100 patients with stage 1–3a chronic kidney disease (CKD) and diabetic nephropathy (Shenglin et al., 2024), combination therapy with finerenone and valsartan significantly reduced serum levels of C-reactive protein (CRP), high-mobility group box protein B1 (HMGB1), and transforming growth factor-beta 1 (TGF-β1). Concurrently, this regimen lowered malondialdehyde (MDA) and reactive oxygen species (ROS) levels while increasing superoxide dismutase (SOD) activity and total antioxidant capacity (T-AOC). Compared to traditional steroidal mineralocorticoid receptor antagonists (s-MRAs) such as spironolactone and eplerenone, finerenone exhibits superior mineralocorticoid receptor selectivity and a more favorable sex hormone-related safety profile; moreover, evidence supporting its benefits for cardiorenal metabolic outcomes is rapidly accumulating (González-Juanatey et al., 2023). Mechanistic investigations in animal models further demonstrated that combined administration of finerenone and exenatide attenuates inflammation and oxidative damage by modulating the NF-κB, PI3K/Akt/mTOR, and NRF2 signaling pathways, thereby exerting potent renoprotective effects in diabetic nephropathy (Kocak et al., 2025). Given its substantial renal and cardiovascular protective efficacy in patients with type 2 diabetes mellitus (T2DM) and CKD, finerenone has been positioned as a “third” or even “fourth” pillar of therapeutic management for diabetic nephropathy, following ACEIs/ARBs and SGLT2 inhibitors (Blazek and Bakris, 2023). These data suggest that, compared with conventional steroidal MRAs, the combination of finerenone with RAAS inhibitors or SGLT2 inhibitors may yield improved clinical outcomes, although the underlying molecular mechanisms and drug interactions remain incompletely elucidated. Collectively, this body of evidence highlights finerenone’s role in advancing the paradigm of RAAS blockade from “broad suppression” toward “selective modulation.”

### 5.5 Novel RAAS inhibitors (e.g., ARNI)

Angiotensin receptor-neprilysin inhibitors (ARNI) have emerged as a pivotal breakthrough in the management of heart failure in recent years. Sacubitril/valsartan, the first-in-class ARNI, is characterized by its dual mechanism of action: valsartan blocks the AT1R to suppress Ang II-mediated inflammation and oxidative stress, while sacubitril inhibits neprilysin to elevate natriuretic peptide levels—these peptides inherently exert vasodilatory effects and inhibit fibrosis and inflammation (Pang et al., 2021).

Animal studies have demonstrated that, compared with valsartan monotherapy, sacubitril/valsartan significantly ameliorates atherosclerosis and inflammation in apolipoprotein E-deficient (apoE^−/−^) mice (Zhang et al., 2019). Similarly, in a doxorubicin-induced rabbit model of heart failure, sacubitril/valsartan treatment mitigates inflammation and oxidative stress, thereby improving cardiac function (Yu et al., 2021). Furthermore, a recent study revealed that sacubitril/valsartan alleviates inflammation and oxidative stress in myocardial tissue of rats with hypertensive heart disease by restoring calcium/calmodulin-dependent protein kinase kinase 2 (CAMKK2) protein expression and activating the AMP-activated protein kinase (AMPK)/protein kinase B (AKT)/glycogen synthase kinase-3β (GSK-3β) signaling pathway (Yang and Li, 2026).

In clinical research, large-scale randomized controlled trials (RCTs) like PARADIGM-HF (Sabe et al., 2015) have established that sacubitril/valsartan confers greater clinical benefits than ACEIs or ARBs monotherapy in the management of chronic heart failure (CHF). A Chinese RCT investigating sequential therapy for acute heart failure (AHF) demonstrated that sacubitril/valsartan treatment significantly reduced serum levels of pro-inflammatory cytokines (including TNF-α and IL-6), while decreasing oxidative stress markers and elevating antioxidant factor levels. Compared with the standard therapy group and recombinant human brain natriuretic peptide (rhBNP) group, the sequential treatment group exhibited superior improvements in left atrial diameter (LAD), left ventricular end-diastolic volume (LVEDV), left ventricular ejection fraction (LVEF), and pulmonary artery pressure (PAP) (Pang et al., 2021). Consistent with these observations, Bunsawat K et al. reported that 3 months of sacubitril/valsartan treatment doubled peripheral vascular function (assessed by brachial artery flow-mediated dilation [FMD]) in patients with heart failure with reduced ejection fraction (HFrEF), enhanced functional capacity (measured by 6-min walk test [6MWT] distance), and reduced pro-inflammatory biomarkers (TNF-α and IL-18) (Bunsawat et al., 1985). These findings indicate that the cardioprotective effects of sacubitril/valsartan in AHF may be partially mediated by its anti-inflammatory and antioxidant activities. Future studies should further explore the therapeutic potential of ARNI in other inflammation- and oxidative stress-related disorders, such as diabetic cardiomyopathy and chronic kidney disease (CKD).

## 6 Evidence of anti-inflammatory and antioxidant applications of RAAS inhibitors in specific disease models or clinical practice

### 6.1 Cardiovascular diseases (e.g., hypertension, heart failure, atherosclerosis)

Hypertension and atherosclerosis represent the primary therapeutic indications for RAAS inhibitors, with inflammation and oxidative stress serving as pivotal contributors to the pathogenesis of both conditions. In patients with hypertension, Ang II-induced oxidative stress drives endothelial dysfunction, vascular smooth muscle cell proliferation, and vascular inflammation—pathophysiological processes that synergistically promote elevated blood pressure and target organ damage (Ekholm and Kahan, 2021; Daiber et al., 2021). By inhibiting RAAS activation, ACEIs and ARBs ameliorate vascular endothelial function, reduce circulating levels of oxidative stress markers, and attenuate the progression of atherosclerosis. A multicenter study involving patients with hypertension complicated by intracranial aneurysms demonstrated a significant association between RAAS inhibitor use and reduced aneurysm rupture risk (OR = 0.490), with risk ratios of 0.559 for ACEI users and 0.414 for ARB users (Zhong et al., 1979). This study provides robust evidence for the cerebrovascular protective effects of RAAS inhibitors. For the prevention of atherosclerosis-related cardiovascular events, large-scale randomized controlled trials have confirmed that ACEIs and ARBs reduce the incidence of major adverse cardiovascular events (MACE) and cardiovascular mortality (Singhania et al., 2020). In patients with acute coronary syndrome, ACEIs are recommended as first-line therapy, whereas ARBs may confer advantages in modulating platelet reactivity (Tscharre et al., 2021). Collectively, current evidence supports the cardiovascular protective effects of RAAS inhibitors in hypertension and atherosclerosis via anti-inflammatory and antioxidant mechanisms (Habib et al., 2025). However, a critical limitation is that none of the aforementioned studies systematically evaluated changes in inflammatory and oxidative stress biomarkers, which hinders the quantification of the clinical significance of these effects.

In addition, heart failure constitutes a key therapeutic indication for RAAS inhibitors, where cardiac inflammatory responses and oxidative stress are recognized as critical drivers of myocardial injury and ventricular remodeling. As cornerstone pharmacotherapy for heart failure with reduced ejection fraction (HFrEF), RAAS inhibitors have been robustly validated by evidence-based medicine to improve patient prognosis (Stolfo and Savarese, 2019). Landmark randomized controlled trials, including CONSENSUS, SOLVD, and CHARM, have consistently demonstrated that ACEIs and ARBs significantly reduce all-cause mortality and cardiovascular mortality in patients with heart failure. Among hospitalized patients with acute heart failure, RAAS inhibitor use confers additional clinical benefits, albeit the supporting evidence is predominantly derived from non-randomized studies (Singhania et al., 2020). Collectively, the clinical benefits of RAAS inhibitors in heart failure patients are well-established, and their cardioprotective effects are likely partially mediated by anti-inflammatory and antioxidant mechanisms (Singhania et al., 2020).

### 6.2 Renal diseases (e.g., diabetic nephropathy, chronic kidney disease)

Chronic kidney disease (CKD) and diabetic kidney disease (DKD) represent additional key therapeutic indications for RAAS inhibitors, with their pathogenesis involving intricate crosstalk between RAAS activation, inflammatory responses, and oxidative stress (Kang and Yoo, 2026). In preclinical studies, various rat models investigating agents including captopril (Azizi-Malekabadi et al., 2020), perindopril (Pastacı Özsobacı et al., 2024), losartan (Qin et al., 2018), and finerenone (González-Juanatey et al., 2023)have consistently demonstrated that RAAS inhibitors exert renoprotective effects across diverse renal disease contexts. These effects are mediated by reducing renal tissue inflammatory infiltrates and oxidative stress markers, as well as enhancing antioxidant enzyme activity.

Elucidating the mechanisms by which RAAS inhibitors attenuate proteinuria in DKD remains a central question in nephrology: does this reduction primarily stem from hemodynamic relief of glomerular hypertension or from non-hemodynamic suppression of podocyte inflammation? Hemodynamically, Ang II elevates intraglomerular pressure via efferent arteriolar vasoconstriction; conversely, RAAS blockade lowers this pressure through efferent vasodilation, thereby reducing protein filtration. Non-hemodynamically, local RAAS activation and hyperglycemia drive glomerulosclerosis and tubulointerstitial fibrosis via upregulation of TGF-β, NADPH oxidase-dependent reactive oxygen species (ROS) generation, and podocyte apoptosis. Notably, the anti-inflammatory and antifibrotic properties of RAAS inhibitors confer renoprotection independent of their systemic blood pressure-lowering effects (Kang and Yoo, 2026). Large randomized controlled trials in DKD have consistently demonstrated that ACEIs and ARBs reduce proteinuria, slow the decline in estimated glomerular filtration rate (eGFR), and delay progression to end-stage renal disease. Furthermore, the magnitude of proteinuria reduction serves as both a mechanistic mediator and a surrogate biomarker for long-term renal outcomes (Koppe and Fouque, 2019). Recent evidence highlights the role of finerenone, a non-steroidal mineralocorticoid receptor antagonists (ns-MRAs), which significantly reduces proteinuria, CKD progression, and cardiovascular risk in patients with type 2 diabetes and chronic kidney disease (CKD) by specifically targeting inflammatory and fibrotic pathways. Even when added to maximally tolerated doses of ACEIs/ARBs, finerenone substantially lowers the incidence of renal and cardiovascular events while exhibiting a lower risk of hyperkalemia compared to steroidal MRAs such as spironolactone (González-Juanatey et al., 2023). Moreover, combination therapy with finerenone and valsartan demonstrates enhanced efficacy and safety profiles (Shenglin et al., 2024). Consequently, finerenone has been established as a “third” or potentially “fourth” pillar of therapeutic management in DKD, following ACEI/ARB and SGLT2 inhibitor therapies (Blazek and Bakris, 2023). However, the utility of RAAS inhibition in advanced CKD remains controversial. While trials such as STOP-ACEi suggest potential cardiovascular benefits from continued RAAS inhibition, clinical apprehension persists regarding its use in late-stage CKD due to heightened risks of hyperkalemia and acute kidney injury (Spencer and Bhandari, 2025). In conclusion, the renoprotective efficacy of RAAS inhibitors in CKD and DKD is well-established, resulting from a synergistic interplay of hemodynamic, anti-inflammatory, antioxidant, and antifibrotic mechanisms. Although these pathways are difficult to disentangle completely, their relative contributions likely vary according to the stage of disease progression (Bahreini et al., 2021).

### 6.3 Other diseases (e.g., metabolic syndrome, pulmonary diseases, neurological disorders, and bone metabolism disorders)

#### 6.3.1 Metabolic syndrome and obesity-related diseases

Accumulating evidence underscores the pivotal role of the renin-angiotensin-aldosterone system (RAAS) in the pathogenesis of insulin resistance. Mechanistically, Ang II precipitates low-grade inflammation via reactive oxygen species (ROS)-mediated signaling, thereby impairing insulin signal transduction. Conversely, RAAS blockade exerts beneficial metabolic effects: ACEIs enhance insulin sensitivity in preclinical models, while ARBs modulate peroxisome proliferator-activated receptor gamma (PPARγ) activity and the hypothalamic-pituitary-adrenal axis (Valente et al., 2021). Clinical data corroborate that RAAS inhibition attenuates the progression of insulin resistance and mitigates associated comorbidities, including type 2 diabetes mellitus (T2DM), dyslipidemia, metabolic syndrome, and obesity (Kalupahana and Moustaid-Moussa, 2024). Furthermore, glucagon-like peptide-1 (GLP-1) receptor agonists and sodium-glucose cotransporter-2 (SGLT2) inhibitors ameliorate chronic inflammation through multifaceted mechanisms, such as cytokine regulation, oxidative stress reduction, modulation of immune cell function and mitochondrial homeostasis, and restoration of endothelial integrity (Blazek and Bakris, 2023). Consequently, the synergistic combination of RAAS inhibitors with GLP-1 receptor agonists offers a dual blockade against oxidative stress, presenting a promising therapeutic strategy for the comprehensive management of metabolic syndrome and T2DM.

Adipose tissue functions not merely as a passive energy reservoir but as a dynamic endocrine organ capable of synthesizing and secreting multiple components of the RAAS system, including angiotensinogen, renin, ACE, AT1R, and ACE2. In obesity, local activation of adipose RAAS drives inflammatory signaling via Ang II-mediated AT1R engagement, which enhances pro-inflammatory cytokine expression in adipocytes and facilitates macrophage infiltration with M1 polarization, thereby establishing a chronic inflammatory microenvironment (Valente et al., 2021). The metabolic benefits conferred by RAAS inhibitors in obesity and metabolic syndrome are partly attributable to their suppression of this local adipose RAAS activity. Furthermore, in metabolic cardiovascular disease, PPARα exerts protective effects by modulating lipid metabolism and attenuating inflammation and oxidative stress, suggesting potential crosstalk between local adipose RAAS and PPARα signaling pathways (Valente et al., 2021). Collectively, these findings underscore the significant contribution of adipose-specific RAAS activation to systemic inflammation; however, the relative magnitude of this effect compared to local RAAS activation in other metabolic tissues requires further quantitative elucidation.

#### 6.3.2 Lung injury-related diseases

In lung injury-associated pathologies, RAAS dysregulation is increasingly recognized as a key driver of pulmonary tissue damage. In LPS-induced acute lung injury models, captopril exhibits dose-dependent protective efficacy (Boskabadi et al., 2019). A retrospective case-control study by Kim et al. (Kim J. et al., 2017) in patients with acute respiratory distress syndrome (ARDS) demonstrated that ACEI or ARB therapy was associated with improved survival rates. Notably, while the RAAS inhibitor cohort exhibited prolonged mechanical ventilation and ICU stays, they showed reduced ICU mortality and superior long-term outcomes. Emerging evidence from COVID-19 cohorts further substantiates the anti-inflammatory potential of RAAS inhibitors (Schnaubelt et al., 2025). Early in the pandemic, the role of ACE2 in SARS-CoV-2 infection sparked debate regarding the “ACE2 downregulation paradox”: whether reduced ACE2 expression confers protection by limiting viral entry receptors or exacerbates tissue injury via disruption of RAAS homeostasis (Yipeng et al., 2020; Fang et al., 2020). Theoretical concerns posited that since SARS-CoV-2 utilizes ACE2 for cellular entry, RAAS inhibition might upregulate ACE2 expression, thereby potentiating viral susceptibility (Yipeng et al., 2020). However, subsequent clinical data refuted this hypothesis, supporting the safety of continued RAAS inhibitor therapy (Ekholm and Kahan, 2021; Cremer et al., 2021). Large-scale meta-analyses corroborated that RAAS inhibitor use correlates with reduced mortality in COVID-19 patients without increasing disease severity, with treated individuals exhibiting attenuated inflammatory markers and improved clinical endpoints (Zhang et al., 2021; Zhang et al., 2022). These findings suggest that RAAS blockade may confer protection through modulation of ACE2 expression or restoration of the Ang II/Ang-(1–7) balance. Collectively, targeting RAAS imbalance via ACEIs/ARBs represents a promising therapeutic strategy for mitigating organ damage, although further investigation is required to elucidate the mechanistic interplay between viral infection, multi-organ dysfunction, and RAAS dysregulation.

#### 6.3.3 Neurological disorders

Within the central nervous system, dysregulation of the brain renin-angiotensin-aldosterone system (RAAS) is intimately linked to the pathogenesis of neurodegenerative disorders, particularly Parkinson’s disease (PD) and Alzheimer’s disease (AD). Preclinical investigations have demonstrated that ACEIs and ARBs possess significant neuroprotective potential (Gouveia et al., 2022). For instance, in models of cisplatin-induced cognitive impairment, captopril mitigated neuroinflammation and oxidative stress, thereby improving learning and memory functions (Mostafa et al., 2025), These protective effects are mediated through the inhibition of NF-κB signaling and its downstream pro-inflammatory cytokines, as well as the suppression of astrocytic activation. In the context of AD, observational data indicate that ARB usage correlates with elevated brain levels of AT4 receptors, reduced oxidative stress, and lower amyloid-beta burden in cognitively normal older adults (Cosarderelioglu et al., 2023), suggesting that ACEIs/ARBs may confer neuroprotection via modulation of the intracerebral RAAS. However, the therapeutic efficacy of these agents is constrained by the blood-brain barrier (BBB); most ACEIs and ARBs exhibit limited CNS penetration, which restricts their ability to directly regulate brain RAAS components (Porel et al., 2025). Notably, comparative studies in AD suggest that ARBs may be more effective than ACEIs in attenuating cognitive decline, a difference potentially attributable to the superior BBB permeability of specific ARBs such as candesartan and telmisartan (Ababei et al., 2023). Leveraging this anti-neuroinflammatory mechanism, telmisartan has been formally selected as an initial intervention candidate within international multi-arm, multi-stage clinical trial platforms for PD (Gonzalez-Robles et al., 2025). Collectively, these findings underscore the potential of ACEI/ARB-mediated inflammation control for drug repurposing in multisystem fibrosis and neurodegenerative diseases. Nevertheless, pharmacokinetic hurdles—specifically the challenge of adequate BBB penetration—remain critical limitations in realizing the full therapeutic potential of local RAAS-targeted therapies in the brain.

#### 6.3.4 Osteoporosis

In osteoporosis, inflammation and oxidative stress imbalance exacerbate bone loss by promoting osteoclast activation and inhibiting osteoblast proliferation. The local skeletal renin-angiotensin-aldosterone system (RAAS), distinct from the systemic RAAS, critically regulates bone metabolism; its dysregulation contributes to osteoporosis and hypocalcemia. Mechanistically, local Ang II–AT1R pathway activation stimulates osteoclasts to secrete TNF-α and IL-6 and generate reactive oxygen species (ROS), accelerating bone resorption. In contrast, ACEIs, ARBs, and mineralocorticoid receptor antagonists (MRAs) mitigate this process through anti-inflammatory and antioxidant effects, suppressing osteoclast activity and fostering osteoblast proliferation to restore bone homeostasis (Haliga et al., 2025; Abuohashish et al., 2017). Clinical evidence from retrospective cohorts shows that long-term ARB therapy in hypertensive patients with osteoporosis significantly slows bone mineral density (BMD) decline and reduces fracture risk compared to other antihypertensives (Gutierrez et al., 2025). A recent meta-analysis confirms an association between ARB use and lower fracture risk, but finds no significant link for ACEIs (Wu et al., 2022). While robust preclinical data and observational studies support the potential benefits of RAAS blockade—likely mediated by reduced oxidative and inflammatory stress—current evidence remains insufficient to classify ACEIs/ARBs as primary osteoporosis treatments due to unresolved controversies regarding their direct bone-protective efficacy in hypertensive populations. However, for patients with both hypertension and skeletal fragility, RAAS inhibitors may serve as modifiable therapeutic factors impacting bone health. Large-scale prospective randomized controlled trials are warranted to determine the definitive role of RAAS inhibitors in osteoporosis prevention and management.

#### 6.3.5 Rheumatoid arthritis (RA)

In rheumatoid arthritis (RA), recent preclinical studies have highlighted the potential therapeutic role of renin-angiotensin-aldosterone system (RAAS) inhibitors. Losartan potassium demonstrated significant anti-arthritic efficacy in RA rat models, characterized by the marked suppression of inflammatory and oxidative stress biomarkers and the modulation of AT1R and AT2R protein expression; this suggests that losartan enhances its therapeutic effect via regulation of the Ang II/AT1R/AT2R axis (Hussein et al., 2025). Similarly, ramipril attenuated inflammation and cartilage degradation in RA rats by inhibiting the JAK-2/STAT-3/RANKL signaling pathway, while concurrently reducing pro-inflammatory Ang II levels and elevating anti-inflammatory Ang 1–7 concentrations (Arab et al., 2022). In an adjuvant-induced arthritis model, aliskiren improved oxidative stress and inflammatory markers through downregulation of the IL-6/JAK2/STAT3 pathway. Aliskiren treatment mitigated MMP-3- and RANKL-mediated joint destruction, reduced iNOS expression, and increased eNOS expression, achieving outcomes comparable to normal controls and methotrexate across most measured parameters (Azouz et al., 2020). Furthermore, a small-scale clinical study indicated that both candesartan and atorvastatin exhibited anti-inflammatory, antioxidant, and immunomodulatory effects, leading to improved clinical status and reduced disease activity in patients with RA (Mostafa et al., 2020). However, clinical evidence regarding the impact of RAAS inhibitors on RA remains inconsistent. A recent cross-sectional study from the Netherlands found no significant association between ACEI or ARB use and disease activity in RA patients (Sluijsmans et al., 2021). Consequently, although preclinical data and limited observational studies suggest promising results for ACEIs, ARBs, and direct renin inhibitors (DRIs), human evidence is sparse and largely observational. The translation of these biological effects into clinically meaningful outcomes in humans remains uncertain (Habib et al., 2025; Moreira et al., 2021).

## 7 Comparative analysis of anti-inflammatory and antioxidant efficacy across five major classes of RAAS inhibitors

### 7.1 Head-to-head comparison of ACEIs and ARBs: controversies regarding CYP2C9 polymorphisms and bradykinin-mediated anti-inflammatory effects

The disparities between ACEIs and ARBs regarding anti-inflammatory, antioxidant, and cardioprotective effects have long been a subject of debate in clinical pharmacology. Although both drug classes exhibit comparable efficacy in blood pressure reduction, several clinical trials and meta-analyses have reported higher cardiovascular mortality and reduced protection against myocardial infarction associated with ARB use compared to ACEIs (Lévy and Mourad, 2022). These clinical discrepancies may stem from fundamental differences in their pharmacological mechanisms: while ACEIs inhibit Ang II formation and concurrently suppress bradykinin degradation, ARBs selectively block the AT1 receptor without affecting bradykinin metabolism. From an anti-inflammatory and antioxidant perspective, the accumulation of bradykinin induced by ACEIs may confer additional benefits, given its inherent properties of reducing inflammation, protecting endothelial function, and inhibiting atherogenesis. Conversely, the relative activation of AT2 and AT4 receptors by ARBs may counterbalance the consequences of AT1 blockade. Furthermore, certain ARBs—particularly telmisartan and candesartan—offer supplementary anti-inflammatory advantages through PPARγ agonism (Haliga et al., 2025).

In a dextran sodium sulfate (DSS)-induced colitis model, enalapril downregulated pro-inflammatory IL-1β expression, while losartan reduced macroscopic scores; notably, the combination of these agents failed to demonstrate synergistic efficacy (Salmenkari et al., 2018). This lack of synergy contrasts with the established benefits of combined therapy in hypertension and heart failure, suggesting distinct pathophysiological mechanisms or differential pharmacological profiles in inflammatory bowel disease (IBD). Furthermore, an observational study involving patients with Crohn’s disease reported that ACE inhibitor use was associated with poorer long-term outcomes, whereas ARB use correlated with reduced corticosteroid requirements (Karime et al., 2024). Similarly, research into neurocognitive disorders has indicated that ARBs exert more potent neuroprotective effects compared to ACE inhibitors (Lundin et al., 2024). In the context of SARS-CoV-2 infection, evidence remains discordant: some studies suggest that ACE inhibitors offer superior protection against severe disease and mortality compared to ARBs (Liu et al., 2023), while other data indicate no significant difference between the two drug classes in improving clinical outcomes (Zhang et al., 2022). Regarding pharmacogenomics, CYP2C9 polymorphisms may modulate ACE inhibitor metabolism and bradykinin-mediated effects, although current evidence in this domain is limited. Collectively, these findings underscore fundamental differences in the anti-inflammatory mechanisms of ACE inhibitors versus ARBs, highlighting the potential role of CYP2C9 genetic variability in influencing individual treatment responses.

### 7.2 Assessing the strength of evidence for the “ARB superiority” hypothesis: subgroup heterogeneity in meta-analyses

The hypothesis that ARBs are superior to ACEIs warrants cautious interpretation, given the significant subgroup heterogeneity revealed by recent meta-analyses. Although Lévy and Mourad’s systematic review noted comparable antihypertensive efficacy between the two classes, multiple clinical trials and meta-analyses have indicated that ARBs may be inferior regarding cardiovascular mortality and myocardial infarction prevention (Lévy and Mourad, 2022). Furthermore, a recent meta-analysis suggested that ACEIs might offer superior renoprotection despite similar reductions in overall cardiovascular risk (Spencer and Bhandari, 2025). These discrepancies likely stem from several key factors:

First, baseline patient characteristics—such as age, comorbidities, and ethnicity—vary significantly across studies, potentially modulating therapeutic responses. Second, the phenomenon of “aldosterone breakthrough,” characterized by plasma aldosterone rebound during long-term RAAS blockade, may compromise efficacy. Here, ACEI-mediated bradykinin accumulation may provide compensatory protective effects that ARBs lack. Third, oncological evidence highlights divergent outcomes: while ACEIs were associated with a 50% reduction in grade ≥2 radiation pneumonitis and lower risks of proctitis (OR: 0.20), hematuria (OR: 0.25), and rectal bleeding (OR: 0.39) in prostate cancer patients, survival data for ARB users remained inconsistent (Liao et al., 2025). This suggests context-dependent effects varying by cancer type and stage. Fourth, regarding immunomodulation, ACEIs demonstrate therapeutic potential in autoimmune myocarditis, colitis, and diabetic nephropathy by attenuating inflammatory cytokines, whereas ARBs’ immunomodulatory effects appear primarily mediated through PPARγ activation pathways (Haliga et al., 2025). n conclusion, the superiority of ARBs over ACEIs is not uniformly supported; rather, the evidence underscores fundamental mechanistic differences influenced by subgroup heterogeneity, study design, and population characteristics. Therefore, broad generalizations are inappropriate. However, for patients intolerant to ACEIs, ARBs remain a clinically viable alternative.

### 7.3 Comparative efficacy of other RAAS inhibitors

In comparative studies of other RAAS inhibitors, Thomas CM et al. (Thomas et al., 2013) demonstrated in a diabetic cardiomyopathy model that aliskiren conferred cardiac protective effects comparable to those of ARBs and ACEIs. However, the three agents exhibited divergent effects on oxidative stress, cytokine profiles, and prorenin receptor (PRR) expression, implying subtle distinctions in their underlying mechanisms of action. Nevertheless, the anti-inflammatory and antioxidant potential evidenced in rodent models cannot be directly extrapolated to clinical outcomes in humans. Furthermore, direct comparative data on the anti-inflammatory and antioxidant efficacy of aliskiren versus other RAAS inhibitors remain limited (Alshahrani, 2020). Notably, finerenone—a non-steroidal mineralocorticoid receptor antagonist (ns-MRA)—has shown superior potential in exerting anti-inflammatory and antifibrotic effects compared to traditional ACEIs/ARBs and Steroidal mineralocorticoid receptor antagonists (s-MRAs), attributable to its unique pharmacological properties, while also mitigating the risk of hyperkalemia (Palanisamy et al., 2022). In preclinical investigations of diabetic retinopathy, both finerenone and perindopril reduced blood pressure and ameliorated retinal vascular pathology; however, finerenone demonstrated greater efficacy in downregulating inflammatory mediators, including VEGF, ICAM-1, and IL-1β (Jerome et al., 2023). Nonetheless, direct comparative data on the anti-inflammatory and antioxidant effects of finerenone versus conventional ACEIs or ARBs in human subjects remain limited. Thus, future research should prioritize head-to-head clinical trials to delineate inter-agent differences among RAAS inhibitors and to establish biomarkers or clinical criteria for identifying patients most likely to benefit from their anti-inflammatory and antioxidant properties.

## 8 Limitations of current evidence and methodological deficiencies

While extensive preclinical evidence supports the anti-inflammatory and antioxidant properties of RAAS inhibitors, significant gaps and methodological limitations persist within the current translational framework. First, the lack of reproducibility between animal models and human clinical trials constitutes a major challenge. The extrapolation of drug dosages from preclinical studies to human applications is inherently uncertain, as experimental doses often far exceed clinically equivalent levels. This discrepancy arises from interspecies variations in receptor affinity, pharmacokinetic/pharmacodynamic (PK/PD) profiles, nonlinear metabolic conversion, and limitations in standard scaling methods. Consequently, these factors compromise the reliability of translating animal findings to human contexts, potentially explaining the attenuated anti-inflammatory and antioxidant effects observed in clinical settings (Boskabadi et al., 2018; Boskabadi et al., 2019). Furthermore, pathological processes in animal models are typically accelerated and oversimplified, failing to capture the complexity and heterogeneity characteristic of human chronic diseases.

Second, investigations into local tissue RAAS activity are constrained by pharmacokinetic discrepancies. Rodents and humans exhibit marked differences in systemic PK parameters (bioavailability, half-life, clearance, and metabolic enzyme expression) as well as tissue-specific bioavailability mechanisms, including local RAAS components, chymase pathways, AT1 receptor subtypes, and transporter expression. These disparities are particularly evident in the context of local Angiotensin II (Ang II) generation: while ACE inhibitors may achieve complete blockade of local Ang II synthesis in rats, the non-ACE-mediated chymase pathway can bypass this inhibition in humans. As a result, high-dose preclinical ACE inhibitor regimens may overestimate their tissue-level anti-inflammatory and antioxidant efficacy relative to actual clinical outcomes (Dell'Italia et al., 2018).

Third, the heterogeneity of oxidative stress biomarkers and the lack of standardized analytical methodologies significantly impede cross-study comparability. Fourth, surrogate endpoints commonly employed in RAAS inhibitor research—including C-reactive protein (CRP), interleukin-6 (IL-6), tumor necrosis factor-alpha (TNF-α), malondialdehyde (MDA), and oxidized low-density lipoprotein (oxLDL)—often demonstrate weak correlations with hard clinical outcomes such as mortality, major adverse cardiovascular events (MACE), or end-stage renal disease (ESRD). Consequently, while most clinical trials prioritize primary endpoints, direct evidence supporting the anti-inflammatory and antioxidant effects of RAAS inhibitors remains sparse, with conclusions frequently derived from preclinical models or indirect inference (Daiber et al., 2021). The absence of systematic biomarker evaluation in many studies further complicates the quantification of these effects’ clinical significance (Mostafa et al., 2020). Fifth, most preclinical investigations lack head-to-head comparisons between combination therapies and monotherapies, limiting the evidence base for multi-target strategies (Karime et al., 2024). Furthermore, the pathophysiological complexity inherent in human patients—characterized by polypharmacy, comorbidities, and inter-individual variability—may substantially modulate therapeutic efficacy (Stolfo and Savarese, 2019).

## 9 Future research directions and perspectives

### 9.1 Development of novel RAAS-Targeting agents

Future research should continue to investigate emerging drug targets within the RAAS system. The ACE2/Angiotensin-(1–7)/Mas axis, as a protective RAAS pathway, has emerged as a key research focus (Mascolo et al., 2020; Wan et al., 2024). ACE2 activators (e.g., Dize), Angiotensin-(1–7) analogs, and Mas receptor agonists hold potential as novel therapeutic agents for the management of inflammation and oxidative stress-related disorders (Wan et al., 2024). Angiotensin II type 2 receptor (AT2R), an alternative receptor subtype for Ang II, exerts biological effects opposing those of AT1R, including promoting vasodilation, inhibiting cell proliferation, and mediating anti-inflammatory actions; thus, the development of highly selective AT2R agonists may provide innovative therapeutic strategies (Mascolo et al., 2020). Moreover, biased ligands represent a frontier in RAAS-targeted therapeutics. Unlike conventional ARBs that non-selectively block all AT1R downstream cascades, biased ligands selectively inhibit deleterious G protein-mediated pathways (e.g., vasoconstriction, aldosterone secretion) while preserving beneficial β-arrestin–mediated signaling (e.g., anti-apoptotic, anti-inflammatory). These agents aim to maintain therapeutic efficacy while minimizing adverse effects and enhancing antioxidant/anti-inflammatory outcomes; however, their safety and long-term benefits require validation via large-scale trials (Rizk et al., 2022).

### 9.2 Optimization of novel combination therapeutic strategies

#### 9.2.1 Synergistic mechanisms of RAAS inhibitors combined with SGLT2 inhibitors and GLP-1 receptor agonists

The combination of RAAS inhibitors with SGLT2i and GLP-1 receptor agonists (GLP-1 RAs) constitutes a rapidly evolving frontier in cardiorenal protection. Mechanistically, SGLT2 inhibitors restore tubuloglomerular feedback and induce afferent arteriolar vasoconstriction, whereas RAAS blockade promotes efferent arteriolar vasodilation; this complementary action synergistically lowers intraglomerular pressure. Both drug classes attenuate oxidative stress, inflammatory signaling, and profibrotic pathways. Notably, SGLT2 inhibition shifts the RAAS equilibrium toward the protective ACE2/Ang-(1–7)/Mas receptor axis across diverse physiological contexts (Aazar et al., 2026). Clinical evidence demonstrates that adding SGLT2i to standard RAAS inhibitor therapy significantly reduces heart failure hospitalizations and slows renal disease progression without compromising safety profiles (Aazar et al., 2026). Furthermore, GLP-1 RAs exhibit nephroprotective efficacy in diabetic kidney disease (DKD), characterized by reductions in albuminuria and attenuation of glomerular filtration rate (GFR) decline. These benefits are mediated through modulation of natriuresis, intrarenal hemodynamics, oxidative stress, endothelial dysfunction, and tubular apoptosis (Braha et al., 2026). Additionally, GLP-1 RAs exert anti-atherogenic effects via improvement of endothelial function and suppression of inflammation and oxidative stress. Current evidence supports the integration of SGLT2i and GLP-1 RAs with RAAS inhibitors as a comprehensive therapeutic strategy for patients at high risk of cardiovascular-kidney-metabolic comorbidities (Braha et al., 2026).

#### 9.2.2 Investigational synergy: combining anti-inflammatory agents (e.g., colchicine) with RAAS inhibitors

The combination of RAAS inhibitors with non-specific anti-inflammatory agents represents a promising avenue for further investigation. Pentoxifylline, an FDA-approved non-selective phosphodiesterase inhibitor, exerts anti-inflammatory effects by suppressing pro-inflammatory cytokine production, as demonstrated in preclinical and clinical studies; small-scale randomized controlled trials (RCTs) and meta-analyses suggest potential therapeutic benefits in DKD (Leehey, 2020). Ongoing large-scale clinical trials are evaluating investigational agents targeting inflammation and oxidative stress, such as bardoxolone methyl and selonsertib, for their efficacy in DKD, where concurrent administration with RAAS inhibitors may confer synergistic protective effects (Leehey, 2020). In the context of Cardiovascular-Kidney-Metabolic (CKM) syndrome, finerenone addresses residual inflammatory and fibrotic risks, exhibiting biological complementarity with SGLT2 inhibitors and thereby serving as a paradigmatic example of rational combination therapy (Kubica et al., 2026). Anti-inflammatory strategies may emerge as novel management approaches in DKD, given that GLP-1 RAs, SGLT2 inhibitors, and RAAS inhibitors collectively possess anti-inflammatory properties, potentially yielding additive or synergistic outcomes upon combined use. However, the implementation of combination regimens necessitates rigorous risk assessment regarding susceptibility to infection and immune-mediated adverse events, particularly among elderly patients and immunocompromised populations.

### 9.3 Translational strategies for anti-inflammatory and antioxidant therapy: from broad-spectrum blockade to precision modulation

In light of the preceding analysis, advancing research on the anti-inflammatory and antioxidant properties of RAAS inhibitors necessitates a paradigm shift from broad-spectrum blockade to precision modulation. This transition is characterized by four core pillars: First, evolving from non-selective RAAS inhibition to tissue-specific targeted interventions. By leveraging nanocarrier delivery systems and biased ligand design, this approach aims to achieve localized drug accumulation in specific tissues and selective regulation of signaling pathways (Habib et al., 2025). Second, transitioning from single-target monotherapy to multi-target combination strategies. Guided by the identification of key nodes within the “inflammation–oxidative stress–RAAS” network topology model, rational combination regimens integrating RAAS inhibitors with SGLT2 inhibitors, GLP-1 receptor agonists, or other anti-inflammatory and antioxidant agents are proposed (Braha et al., 2026). Third, shifting from empirical prescribing to biomarker-stratified personalized medicine. This involves integrating genotypic data (e.g., polymorphisms in AGT, ACE, and AT1R) with inflammatory and oxidative stress biomarkers (such as plasma CRP, IL-6, TNF-α, MDA, SOD, and 8-isoprostane levels) to optimize drug selection and dosage adjustment (Daiber et al., 2021). Fourth, expanding the therapeutic focus from hemodynamic effects alone to include quantitative assessment of anti-inflammatory and antioxidant outcomes. Establishing comprehensive efficacy evaluation metrics that capture the pleiotropic actions of RAAS inhibitors is essential (Seidu et al., 2022). The integrated implementation of these strategies holds the potential to preserve the cardiovascular and renal protective benefits of RAAS inhibitors while maximizing their anti-inflammatory and antioxidant efficacy and minimizing adverse events, thereby facilitating the clinical translation of precision anti-inflammatory and antioxidant therapy.

### 9.4 Design of large-scale, multicenter clinical trials for specific classical and off-label indications

In light of the limitations inherent in existing evidence, the execution of large-scale, multicenter RCTs specifically designed to evaluate the modulatory effects of RAAS inhibitors on inflammatory and oxidative stress pathways is imperative for facilitating their expansion into novel therapeutic indications. This is particularly pertinent for emerging agents such as finerenone and ARNIs. Trial design should incorporate the following critical elements: First, participant selection: Future investigations should implement a precision enrollment strategy grounded in phenotypic and genotypic stratification, prioritizing the inclusion of individuals exhibiting high-inflammation and high-oxidative-stress phenotypes. Second, intervention protocols: The study design should include head-to-head comparisons among distinct subtypes of RAAS inhibitors. Furthermore, dose-response assessments are required to delineate optimal benefit-risk ratios and optimize treatment durations. Third, endpoint assessment: A balanced emphasis must be placed on both primary clinical endpoints (comprising all-cause mortality, myocardial infarction, stroke, and end-stage renal disease) and surrogate biomarker endpoints. Specifically, an Integrated Oxidative Stress and Inflammation Score (IOSS) should be employed, incorporating clinically validated markers including hs-CRP, IL-6, TNF-α, MDA, 8-isoprostane, superoxide dismutase (SOD), catalase (CAT), and glutathione (GSH). Establishing quantitative correlations between these surrogate indices and hard clinical outcomes is essential to validate whether the anti-inflammatory and antioxidant mechanisms of RAAS inhibitors translate into tangible clinical benefits. Fourth, standardization of biomarker assays: To ensure reproducibility and comparability across centers, biomarker detection must adhere to uniform standard operating procedures (SOPs), rigorous multicenter quality control measures, and calibration against certified reference materials, complemented by dynamic longitudinal monitoring. Finally, subgroup analyses and complementary validation: Pre-specified subgroup analyses should be conducted to elucidate the moderating effects of demographic variables (sex, age, ethnicity), baseline comorbidities, and genetic polymorphisms on therapeutic efficacy. Additionally, integrating real-world data (RWD) with RCT findings will provide robust, complementary external validation of the results.

## 10 Conclusion

In summary, the bidirectional modulation of inflammatory cascades and oxidative stress pathways underpins the pleiotropic multi-organ protective effects of RAAS inhibitors, extending beyond their hemodynamic control to safeguard cardiac, renal, and pulmonary function. Synthesizing current preclinical and clinical data reveals that the five distinct classes of RAAS inhibitors exhibit heterogeneous anti-inflammatory and antioxidant efficacies across various pathological contexts. These class-specific distinctions are largely governed by pharmacokinetic properties—such as tissue penetration and receptor subtype selectivity—as well as the degree of activation of the protective ACE2/Ang-(1–7)/Mas axis and local tissue RAAS expression profiles. Consequently, in non-classical indications such as COPD, Alzheimer’s disease (AD), rheumatoid arthritis (RA), and osteoporosis, these immunomodulatory and redox-regulating properties position RAAS inhibitors as promising adjunctive therapeutic options. Therefore, clinical paradigms should evolve from a strictly blood pressure-centric approach to a holistic model that integrates the assessment of target-organ inflammation and oxidative stress burden into comprehensive treatment goals.

The evolution of RAAS inhibitors from simple antihypertensive agents to recognized broad-spectrum anti-inflammatory, antioxidant, and organ-protective therapeutics exemplifies the paradigm of translational medicine. However, bridging the gap between molecular mechanisms and clinical utility remains fraught with challenges. Although preclinical data consistently demonstrate their anti-inflammatory and antioxidant properties, translating these findings into meaningful improvements in human clinical outcomes is constrained by interspecies variability, incomplete suppression of tissue-localized RAAS activity, and safety concerns associated with dual or multiple RAAS blockade. Furthermore, critical gaps persist within the current evidence chain, significantly limiting its robustness: uncertainty in dose extrapolation from preclinical models, poor correlation between surrogate endpoints and hard clinical outcomes, substantial heterogeneity across study designs, publication bias, and conflicts of interest all compromise the overall quality of the available evidence.

Future research must prioritize the design of large-scale, rigorous, and highly representative RCTs within a precision medicine framework, guided by disease-specific mechanisms and individualized biomarkers. Concurrently, there is an urgent need to standardize detection methodologies and ensure the comprehensive publication of negative results to mitigate reporting bias. Only through these measures can the molecular evidence underlying the anti-inflammatory and antioxidant effects of RAAS inhibitors be effectively translated into clinical interventions that improve hard endpoints, thereby completing the translational loop from bench to bedside. This paradigm shift aims to evolve chronic disease management from mere “blood pressure control” to “precision target organ protection.”

## Glossary

RAAS: Renin-angiotensin-aldosterone system
ACEI: angiotensin-converting enzyme inhibitor
ARB: angiotensin receptor blocker
ARNI: angiotensin receptor-neprilysin inhibitor
Ang I: angiotensin I
Ang II: angiotensin II
AT1R: angiotensin II type 1 receptor
AT2R: angiotensin II type 2 receptor
ACE: angiotensin-converting enzyme
CRP: C-reactive protein
CKD: chronic kidney disease
DKD: diabetic kidney disease
DRI: direct renin inhibitors
IL-1β: interleukin-1β
IL-6: interleukin-6
MasR: Mas receptor
MRA: mineralocorticoid receptor antagonists
MCP-1: monocyte chemoattractant protein-1
MAPK: mitogen-activated protein kinase
MrgD: mas-related G protein-coupled receptor D
MDA: malondialdehyde
NF-κB: nuclear factor κB
Nrf2: nuclear factor erythroid 2-related factor 2
PPARγ: peroxisome proliferator-activated receptor gamma
RANKL: nuclear factor-κb ligand
RA: rheumatoid arthritis
ROS: reactive oxygen species
SOD: superoxide dismutase
T2DM: type 2 diabetes mellitus
TGF-β: transforming growth factor β
TNF-α: tumor necrosis factor-α
